# CXCR3 signaling in glial cells ameliorates experimental autoimmune encephalomyelitis by restraining the generation of a pro-Th17 cytokine milieu and reducing CNS-infiltrating Th17 cells

**DOI:** 10.1186/s12974-016-0536-4

**Published:** 2016-04-11

**Authors:** Chen-Yen Chung, Fang Liao

**Affiliations:** Institute of Biomedical Sciences, Academia Sinica, Taipei, 11529 Taiwan

**Keywords:** CXCR3, Experimental autoimmune encephalomyelitis, Th17, Glial cells

## Abstract

**Background:**

Experimental autoimmune encephalomyelitis (EAE) is a mouse model of multiple sclerosis (MS). It has been shown that Th17 cells are critical for EAE pathogenesis. Mice lacking CXCR3 develop aggravated EAE compared with wild-type (WT) mice. This study investigated the effect of CXCR3 on Th17 expansion during EAE and further addressed the underlying mechanism.

**Methods:**

Both active EAE and adoptive-transfer EAE experiments were employed for studying EAE pathogenesis in WT and CXCR3^−/−^ mice. Demyelination and leukocyte infiltration in the spinal cord of mice were analyzed by luxol fast blue staining and flow cytometry analysis, respectively. Glial cells expressing CXCR3 in the spinal cord were analyzed by immunofluorescence staining. Cytokine and chemokine levels in the spinal cord were analyzed using quantitative real-time PCR and enzyme-linked immunosorbent assay (ELISA). The glial cell line U87MG was employed for studying the CXCR3 signaling-mediated mechanism regulating Th17 expansion.

**Results:**

CXCR3^−/−^ mice exhibited more severe EAE and had significantly increased central nervous system (CNS)-infiltrating Th17 cells compared with WT mice. Adoptive-transfer experiments showed that CXCR3^−/−^ recipient mice that received Th17 cells polarized from splenocytes of myelin oligodendrocyte glycoprotein (MOG)-immunized CXCR3^−/−^ mice or MOG-immunized WT mice always developed more severe EAE and had significantly increased CNS-infiltrating Th17 cells compared with WT recipient mice that received Th17 cells from the same origin. Furthermore, during EAE, the number of activated glial cells was increased in the CNS of MOG-immunized CXCR3^−/−^ mice, and CXCR3-deficient glial cells expressed increased levels of cytokine genes required for Th17 expansion and recruitment. Finally, we found that extracellular signal-regulated kinase (ERK) activation elicited by CXCR3 signaling in U87MG cells attenuated the activation of NF-κB, a key transcription factor critical for the induction of IL-23 and CCL20, which are required for Th17 cell expansion and recruitment, respectively.

**Conclusions:**

This study demonstrates a previously unrecognized role of CXCR3 signaling in glial cells in negatively regulating Th17 cell expansion during EAE. Our results demonstrate that, in addition to its well-known role in the recruitment of immune cells, CXCR3 in CNS glial cells plays a critical role in restraining the pro-Th17 cytokine/chemokine milieu during EAE, thereby diminishing Th17 cell expansion in the CNS and suppressing disease development.

**Electronic supplementary material:**

The online version of this article (doi:10.1186/s12974-016-0536-4) contains supplementary material, which is available to authorized users.

## Background

Multiple sclerosis (MS) is a chronic demyelinating inflammatory disease of the central nervous system (CNS) that leads to neurological impairment and, subsequently, disability in the majority of patients [1]. A mouse model of MS is the experimental autoimmune encephalomyelitis (EAE) [2]. The pathogenesis of MS and EAE results from infiltration of the CNS by myelin-specific autoreactive T cells, which attack the myelin sheath. The Th1 subset of T cells, driven by stimulation with interleukin-12 (IL-12), composed of p40 and p35 subunits, has traditionally been considered essential for the pathogenesis of EAE because mice lacking the p40 subunit are resistant to EAE [3, 4]. However, in contrast to mice lacking the p40 subunit, mice lacking the p35 subunit are highly susceptible to the induction of EAE, establishing that IL-12 p70 may not be essential for the development of EAE [5]. Notably, the p40 subunit of IL-12 is shared by IL-23, composed of p40 and p19 subunits [6], and mice lacking either of these subunits are resistant to EAE [7], suggesting that IL-23 is central to the development of EAE. Because IL-23 is critical for the maintenance, survival, and proliferation of Th17 cells [8, 9], this T cell subtype has since been thought to play a pivotal role in the development of EAE. Consistent with this idea, mice lacking IL-17 or RORγt, a transcription factor for Th17 cells, and mice treated with an IL-17 blocking antibody are less susceptible to EAE [9–11]. Importantly, autoreactive Th17 cells preferentially expressing CCR6 have been shown to trigger the initial inflammation by entering the CNS through the choroid plexus via a CCR6-CCL20-dependent mechanism and further infiltrating the CNS parenchyma by a CCR6-independent mechanism [12]. In accord with these findings, studies in human patients have shown that Th17 cells are involved in MS [13, 14].

The C-X-C motif chemokine receptor CXCR3 is mainly expressed on effector leukocytes, including CD4^+^ and CD8^+^ T cells, natural killer (NK) cells, NKT cells, and subsets of B cells [15]. CXCR3 and its ligands, CXCL9, CXCL10, and CXCL11, play a pivotal role in the regulation of inflammatory and infectious diseases, in which CXCR3-bearing effector leukocytes are recruited to the sites of inflammation or infection [16]. In particular, CXCR3 and its ligands play crucial roles in mediating inflammatory diseases of the CNS [17, 18] and in controlling neurotropic viral infection in the CNS [19–24]. Although CXCR3 is mainly expressed on cells of hematopoietic origin, CXCR3 and its ligands have been detected in the CNS [25–28]. Thus, it is expected that CXCR3 and its ligands would not only be critical for the recruitment of effector leukocytes but could also be important for the regulation of CNS physiology during CNS inflammation and infection.

Interestingly, studies on the role of CXCR3 in EAE have yielded quite controversial findings [29–31]. Two reports have shown that CXCR3^−/−^ mice are susceptible to EAE [29, 30]; however, different underlying mechanisms were proposed. Liu et al. showed that CNS tissue damage is increased in CXCR3^−/−^ mice in association with decreased production of interferon (IFN)-γ and iNOS (inducible nitric oxide synthase) [29], whereas Muller et al. showed that the number of Foxp3^+^ regulatory T cells was reduced in the CNS of CXCR3^−/−^ mice [30]. In contrast, several studies reported that blockade or genetic deficiency of either CXCR3 or its primary ligands had no impact [31] or detrimental effects on the manifestations of EAE [31–34]. Thus, the role of CXCR3 in EAE remains elusive. In this study, we demonstrated that CXCR3^−/−^ mice are more susceptible to EAE. They also exhibit an increase in CNS-infiltrating Th17 cells during EAE, which reflects increased expansion of Th17 cells owing to increased numbers of glial cells in the CNS and an enhanced pro-Th17 cytokine/chemokine milieu produced by CXCR3-deficient CNS glial cells. Notably, our study is the first to demonstrate a critical role of CXCR3 in CNS glial cells rather than in CNS-infiltrating T cells in the pathogenesis of EAE.

## Methods

### Mice

The generation of CXCR3^−/−^ mice was described previously [35]. The mice used in these experiments were backcrossed to C57BL/6 for ten generations. For adoptive-transfer EAE experiments, littermates of C57BL/6 wild-type (WT) or CXCR3^−/−^ mice were used as donors and Thy1.1^+/+^C57BL/6 and Thy1.1^+/+^CXCR3^−/−^ mice were used as recipients. All mice were bred under specific pathogen-free conditions at the animal center of the Institute of Biomedical Sciences (Academia Sinica, Taipei, Taiwan). All animal experiments were approved by the Institutional Animal Care and Utilization Committee at Academia Sinica and were performed in accordance with institutional guidelines.

### Cell line

The glioblastoma cell line U87MG was obtained from Dr. Pang-Hsien Tu (Academia Sinica, Taipei, Taiwan) and cultured in Dulbecco’s modified Eagle medium (DMEM; Life Technologies, Carlsbad, CA) supplemented with 10 % fetal bovine serum (FBS; Life Technologies), 2 mM l-glutamine, 1 mM sodium pyruvate, 10 mM HEPES (pH 7.0), and 0.1 mM nonessential amino acids at 37 °C in a humidified 5 % CO_2_ atmosphere.

### Induction of EAE

Mice (8–12 weeks old) were immunized subcutaneously in the hind limbs on day 0 with 100 μg of myelin oligodendrocyte glycoprotein (MOG)_35–55_ peptide (MEVGWYRSPFSRVVHLYRNGK; MDBio, Taipei, Taiwan) in complete Freund’s adjuvant (CFA) containing 400 μg of heat-killed *Mycobacterium tuberculosis* H37RA (Sigma-Aldrich, St. Louis, MO). Two hundred nanograms of pertussis toxin (PTX) (List Biological Laboratories, Campbell, CA) was injected intraperitoneally on days 0 and 2. Mice were graded daily on a clinical scale of 0–6: 0, no sign; 0.5, partially flaccid tail; 1, tail paralysis; 2, impaired righting reflex or gait; 3, partial hind limb paralysis; 4, total hind limb paralysis; 5, hind limb paralysis with partial front limb weakness; and 6, moribundity or death.

### H&E and LFB staining

Mice were anesthetized and intracardially perfused with saline followed by 4 % paraformaldehyde in phosphate-buffered saline (PBS). Spinal cords were embedded in paraffin and then cut into 5-μm-thick transverse sections. Sections were deparaffinized, hydrated, and stained with hematoxylin and eosin (H&E) and luxol fast blue (LFB). For LFB staining, sections were incubated with LFB solution at 60 °C overnight and then washed sequentially with 95 % ethanol, water, 0.1 % lithium carbonate solution, 70 % ethanol, and water. The sections were then dehydrated with ethanol, rinsed with xylene, and mounted. In some experiments, sections stained with LFB were counterstained with cresyl violet.

### Confocal microscopy

Sections were deparaffinized and hydrated with an ethanol series (100, 95, 90, 80, and 70 %, sequentially). The sections were then boiled in retrieval solution (Dako, Glostrup, Denmark) for 40 min and cooled to room temperature. After blocking with PBS containing 5 % bovine serum albumen (BSA) and 0.2 % Tween-20 at room temperature for 30 min, sections were incubated at 4 °C overnight with a primary antibody to Iba1 (Wako, Osaka, Japan). The sections were then washed and incubated with species-specific secondary antibody conjugated with Alexa Fluor 568 (Life Technologies) together with Alexa Fluor 488-conjugated anti-glial fibrillary acidic protein (GFAP; clone GA5; eBioscience, San Diego, CA) at 4 °C overnight. The sections were washed with PBS, mounted with fluorescence mounting medium (Dako) containing 1 μg/ml of DAPI (4’,6-diamidino-2-phenylindole), and observed by confocal microscopy (LSM 700 system with a Plan Apochromat ×10 objective; Carl Zeiss, Oberkochen, Germany). Images were acquired with ZEN software (Carl Zeiss), and data were analyzed using MetaMorph software (SPOT Imaging Solutions, Sterling Heights, MI).

To perform immunofluorescence staining of CXCR3 expression on glial cells in the spinal cord, spinal cords were embedded and frozen in OCT (Sakura, Alphen an den Rijn, Netherlands). Ten-micrometer transverse sections were warmed at room temperature for 10 min, fixed in ice-cold acetone for 5 min, and air-dried for 10 min. Sections were then washed with PBST (0.05 % Tween-20 in PBS) and blocked with PBST containing 5 % BSA for 1 h. After blocking, sections were stained with hamster anti-mouse CXCR3 (clone CXCR3-173; BioLegend, San Diego, CA) along with anti-GFAP-Alexa Fluor 647 (clone 2E1.E9; BioLegend) for detecting the expression of CXCR3 on astrocytes or with rabbit anti-mouse Iba1 (Wako) for detecting the expression of CXCR3 on microglia. After 4-h incubation, sections for detecting CXCR3 on microglia were washed with PBST and further incubated with biotin-conjugated goat anti-hamster IgG (clone Poly4055; BioLegend) along with goat anti-rabbit Alexa Fluor 568 (Thermo Fisher Scientific) for 2 h at room temperature followed by washing with PBST. All sections were then incubated with Alexa Fluor 488-conjugated streptavidin (BioLegend) for 2 h at room temperature. Finally, sections were incubated with 0.1 % Suden black (Sigma-Aldrich) for 10 min, washed, mounted, and inspected using confocal microscopy (LSM 700 system with a Plan Apochromat ×40 objective). To perform immunofluorescence staining of IL-17 expression in the spinal cord during EAE, frozen sections were prepared, fixed, blocked as above, and followed by sequential staining with rat anti-mouse CD4 (BD Bioscience, Franklin Lake, NJ) and goat anti-mouse IL-17 (Santa Cruz Biotechnology, Dallas, TX), donkey anti-goat Alexa Fluor 488 (Thermo Fisher Scientific, Rockford, IL), biotin-conjugated donkey anti-rat IgG (Abcam, Cambridge, MA), and finally Alexa Fluor 555-conjugated streptavidin (Thermo Fisher). The sections were inspected using confocal microscopy (LSM 700 system with a Plan Apochromat ×40 objective).

### Th17 polarization and adoptive transfer

For adoptive-transfer EAE experiments, donor mice were immunized as described above except without injection of PTX. Ten days after immunization, mice were sacrificed, and splenocytes were harvested and subjected to Th17-polarizing culture. Cells (4 × 10^6^ cell/ml) were cultured at 37 °C in a humidified 5 % CO_2_ atmosphere for 72 h in RPMI 1640 medium (Life Technologies) supplemented with 10 % FBS (Thermo Scientific, Rockford, IL); 2 mM l-glutamine; 50 μM 2-mercaptoethanol (Merck, Whitehouse Station, NJ); 100 U/ml of penicillin and 100 μg/ml of streptomycin; 10 μg/ml of MOG_35–55_; Th17-polarizing cytokines, including IL-6 (20 ng/ml; R&D Systems, Minneapolis, MN), IL-23 (20 ng/ml; R&D Systems), and transforming growth factor (TGF)-*β* (5 ng/ml; PeproTech, Rocky Hill, NJ); and neutralizing antibodies against various cytokines (eBioscience), including IL-4 (10 μg/ml), IFN-γ (10 μg/ml), and IL-2 (10 μg/ml). The cultured cells were harvested, and CD4^+^ T cells were purified using magnetic-activated cell-sorting (MACS) beads (Miltenyi Biotec, Auburn, CA). Th17 cells (3 × 10^5^ cells per mouse for analysis of survival rate; 2 × 10^5^ cells per mouse for cell cycle analyses) were intravenously injected into recipient mice that had been irradiated with a dose of 4 Gy. Recipient mice were immunized with 10 μg of MOG_35**–**55_ peptide in CFA containing 50 μg of *M. tuberculosis* and received 200 ng of PTX intraperitoneally on days 0 and day 2. Mice were monitored daily, and clinical scores were graded.

### Isolation of CNS-infiltrating leukocytes

Spinal cords were collected on days 0, 10, and 15 post-immunization for active EAE and on day 12 post-immunization for adoptive-transfer EAE. The spinal cord was excised, minced, and subjected to enzymatic digestion by incubating tissues in 1 ml of digestion buffer containing serum-free RPMI medium supplemented with 0.5 mg/ml of collagenase IV (Sigma-Aldrich) and 1000 U/ml of DNase I (Calbiotech, Spring Valley, CA) for 30 min at 37 °C. Cells were washed once with PBS containing 5 % FBS, collected by centrifugation at 340×*g*, and resuspended in 5 ml of 30 % Percoll solution (GE Healthcare Life Sciences, Piscataway, NJ). The 30 % Percoll homogenate mix was layered over 70 % Percoll followed by centrifugation at 2000×*g* for 20 min at room temperature. Leukocytes were collected from the 30 %/70 % Percoll interface and, after washing three times with complete medium, were ready for immunostaining and cell cycle experiments.

### Flow cytometry analysis

For intracellular cytokine staining, cells were stimulated with 20 ng/ml of phorbol 12-myristate 13-acetate (PMA; Sigma-Aldrich) and 1 μM ionomycin (Sigma-Aldrich) in the presence of 10 μg/ml of brefeldin A (BioLegend) for 4 h at 37 °C. Cells were then harvested, washed with fluorescence-activated cell-sorting (FACS) buffer (HBSS containing 1 % FBS and HEPES, pH 7.0), and incubated with anti-CD16/CD32 (2.4G2, BD Bioscience) at 4 °C for 15 min followed by surface staining with anti-CD4-APC-Cy7 or anti-CD4-APC (RM4-5; BioLegend) at 4 °C for 15 min. After two washes with PBS, cells were stained with fixable viability dye conjugated with eFluor 780 (eBioscience) at 4 °C for 15 min, washed with PBS buffer, and fixed with 2 % paraformaldehyde at 4 °C. After a 1-h fixation, cells were washed with intracellular staining (IC) buffer containing 1 % BSA and 0.1 % saponin (Sigma-Aldrich) in PBS at 4 °C. Cells were then incubated with 2 % normal rat serum (eBioscience) in IC buffer for 30 min followed by intracellular staining with anti-IL-17-PerCp-Cy5.5 (TC11-18H10.1; BioLegend) and anti-IFN-γ-PE (XMG1.2; BioLegend) at room temperature for 30 min. After two washes with FACS buffer, cells were subjected to FACS analysis. For surface staining of CXCR3 expression on U87MG cells, cells were stained with anti-CXCR3-APC (CXCR3-173; BioLegend) followed by FACS analysis. FACS analyses were performed using the LSRII system (BD Biosciences), and data were analyzed using FlowJo software (Tree Star, Ashland, OR).

### Cell cycle analysis of CNS-infiltrating leukocytes

For cell cycle analysis of CNS-infiltrating cells, mice were intraperitoneally injected with 1 mg of BrdU (BD Biosciences) on day 11 post-immunization with MOG. Twenty-four hours after BrdU injection, CNS-infiltrating leukocytes were isolated, cultured for 4 h, and subjected to cell cycle analysis using a BrdU Flow Kit according to the manufacturer’s instructions (BD Biosciences). Briefly, cells were harvested, immunostained with anti-CD4-APC-Cy7, washed and fixed with Cytofix/Cytoperm buffer at 4 °C for 30 min, washed with Perm/Wash buffer, resuspended in Cytoperm Plus buffer at 4 °C for 10 min, and washed again. Cells were fixed once more with Cytofix/Cytoperm buffer at room temperature for 5 min followed by several washes. Cells were then treated with DNase (300 μg/ml) for 1 h at 37 °C, washed, and stained with anti-IL-17-PerCp-Cy5.5, anti-IFN-γ-PE, and anti-BrdU-FITC for 20 min at 37 °C. Finally, cells were washed, resuspended in 1 ml of FACS buffer containing 7-AAD (7-aminoactinomycin D), and subjected to FACS analysis.

### Generation of primary microglia and astrocytes from neonatal mice

The cerebral cortex was dissected from a neonatal mouse (1-day old) brain, placed in HBSS containing 100 U/ml of penicillin and 100 μg/ml of streptomycin, and mechanically disrupted by gently pipetting. Cells were cultured in DMEM supplemented with 10 % FBS (Life Technologies), 2 mM l-glutamine, 100 U/ml of penicillin, 100 μg/ml of streptomycin, OPI media supplement (1 mM oxaloacetate, 0.45 mM pyruvate, 0.2 U/ml of insulin; Sigma-Aldrich), and 0.5 ng/ml of granulocyte/macrophage-colony stimulating factor (GM-CSF; PeproTech) in a 75T flask coated with poly-l-lysine (Sigma-Aldrich) at 37 °C in a humidified 5 % CO_2_ atmosphere. The medium was completely replaced with fresh medium on day 1, and half of the medium was replaced with fresh medium on days 4, 7, and 10. On day 12, microglia were obtained by vigorously slapping the flask to dislodge microglia attached to the astrocyte monolayer. Adherent astrocytes were subjected to trypsin-EDTA digestion, washed, and collected. The purity of microglia and astrocytes was determined by surface staining with anti-CD11b (clone M1/70; eBioscience) for microglia and by intracellular staining with anti-GFAP (clone GA5; eBioscience) for astrocytes. Both cell preparations were >90 % pure. Isolated microglia and astrocytes (2 × 10^5^ cells/ml) were plated in DMEM supplemented with 10 % FBS, 2 mM l-glutamine, 100 U/ml of penicillin, 100 μg/ml of streptomycin and OPI, and cultured overnight, followed by incubation with or without lipopolysaccharide (LPS) (100 ng/ml; Sigma-Aldrich) for 24 h. Culture supernatants were collected for analysis of cytokines by enzyme-linked immunosorbent assay (ELISA).

### ELISA

The levels of IL-23p19 (eBioscience) and CCL20 (R&D Systems) in culture supernatants were measured by ELISA according to the manufacturers’ instructions.

### RT-PCR

Total RNA was extracted from the spinal cord using the TRIzol reagent (Life Technologies) according to the manufacturer’s instructions. Total RNA was reversed transcribed into complementary DNA (cDNA) using SuperScript III reverse transcriptase (Life Technologies) and random hexamer primers according to the manufacturer’s instructions. The cDNA was subjected to quantitative real-time reverse transcription polymerase chain reaction (RT-qPCR) using TaqMan Gene Expression Assays (Applied Biosystems, Foster City, CA) for analysis of the target genes, *IL-23p19*, *CCL20*, *IL-17*, *GM-CSF*, *CXCL9*, and *CXCL10*; *GAPDH* (glyceraldehyde-3-phosphate dehydrogenase) was used as an internal control. The thermal cycling protocol entailed initial heating at 95 °C for 10 min followed by 40 cycles of 95 °C for 1 min and 60 °C for 1 min. The resultant PCR products were measured using an ABI prism 7500 Sequence Detection System (Applied Biosystems). The levels of target messenger RNAs (mRNAs), normalized to the level of *GAPDH*, were analyzed using the ∆∆Ct method.

### Western blotting

U87MG cells (10^6^) were serum-starved for 6 h in fasting medium (DMEM supplemented with 0.5 % BSA, 2 mM l-glutamine, 1 mM sodium pyruvate, 10 mM HEPES pH 7.0, and 0.1 mM nonessential amino acids) followed by treatment with 100 ng/ml of CXCL10 (PeproTech) prior to stimulation with or without 100 ng/ml of IL-17 (R&D Systems). Cells were lysed in a lysis buffer containing 25 mM Tris (pH 7.4), 150 mM NaCl, 1 mM EDTA, 1 mM EGTA, 1 % Triton X-100, protease inhibitor cocktail (EMD Millipore, Billerica, MA), and phosphatase inhibitor cocktail (Sigma-Aldrich). Proteins in cell lysates were resolved by sodium dodecyl sulfate-polyacrylamide gel electrophoresis (SDS-PAGE) on 10 % gels followed by Western blot analysis using antibodies against extracellular signal-regulated kinase (ERK), phospho-ERK, IκB, phospho-IκB, NF-κB p65, poly-(ADP-ribose) polymerase (PARP; all from Cell Signaling Technology, Beverly, MA), and α-tubulin (Abcam).

### Subcellular fractionation

Cells were harvested, washed with PBS, pelleted, and resuspended in hypotonic buffer (10 mM HEPES pH 7.9, 1.5 mM MgCl_2_, 10 mM KCl, 0.5 mM DTT, protease inhibitor cocktail) and incubated on ice for 15 min. After incubation, 10 % NP-40 was added to the cell suspensions (1/16, *v*/*v*) and mixed by vortexing for 10 s. The lysates were then centrifuged at 12,000×*g* at 4 °C for 5 min to pellet nuclei, and supernatants were collected as the cytoplasmic fraction. The nuclear pellets were washed twice with hypotonic buffer and then resuspended in hypertonic buffer (20 mM HEPES pH 7.9, 1.5 mM MgCl_2_, 420 mM NaCl, 25 % glycerol, protease inhibitor cocktail). Nuclear lysates were incubated at 4 °C for 15 min followed by centrifugation at 12,000×*g* for 5 min at 4 °C. Supernatants were collected as the nuclear fraction. Both cytoplasmic and nuclear fractions were subjected to SDS-PAGE followed by Western blot analysis.

### Statistical analysis

Data are presented as means ± SEM. Because the sample size is small (<30 samples) and the data is not normally distributed evaluated by Shapiro–Wilk test, we used Mann–Whitney *U* test to evaluate the significance of differences between two experimental results. The Log-rank test was used for the analysis of survival rates. A *P* < 0.05 was considered statistically significant.

## Results

### CXCR3^−/−^ mice develop more severe EAE

**Fig. 1:**
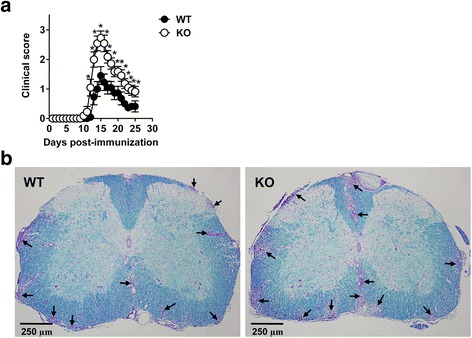
CXCR3^−/−^ mice develop more severe EAE. EAE was induced in WT (*n* = 11) and CXCR3^−/−^ (*n* = 11) mice by immunizing with MOG/CFA. **a** Clinical scores of WT and CXCR3^−/−^ mice at various times after immunization are shown. Data are representative of six independent experiments (*P < 0.05). **b** Sections of the lumbar spinal cord (original magnification ×100) collected from WT and CXCR3^−/−^ mice at the peak of disease (day 15) were fixed and subjected to LFB staining followed by counterstaining with cresyl violet for detection of demyelination (*blue*) and inflammation (*purple*). *Arrows* indicate infiltrating leukocytes. *n* stands for the number of mice

To examine the impact of CXCR3 on the development of EAE, we induced EAE in WT and CXCR3^−/−^ mice and determined the kinetics of disease onset and severity. Both WT and CXCR3^−/−^ mice developed a monophasic disease characterized by ascending paralysis from day 12 to day 15 after immunization followed by a gradual recovery (Fig. 1a). CXCR3^−/−^ mice had slightly earlier disease onset (day 12) compared with WT mice (day 13.8), and the overall disease development was significantly more severe in CXCR3^−/−^ mice than in WT mice (*P* < 0.05, Fig. 1a). We further performed LFB staining to examine demyelinating lesions and leukocyte infiltration in the spinal cord of WT and CXCR3^−/−^ mice. Equivalent sections of lumbar spinal cords from WT and CXCR3^−/−^ were compared, and CXCR3^−/−^ mice exhibited substantial demyelination accompanied by an increase in inflammation in the spinal cord compared with WT mice (Fig. 1b). The comparison of demyelination and inflammation in serial sections from lumbar spinal cords of WT and CXCR3^−/−^ mice is also shown in Additional file 1: Figure S1, and sections of the spinal cords from CXCR3^−/−^ mice consistently showed more severe demyelination and inflammation as compared with WT mice.

### CXCR3^−/−^ mice exhibit significantly increased CNS-infiltrating Th17 cells during EAE

**Fig. 2:**
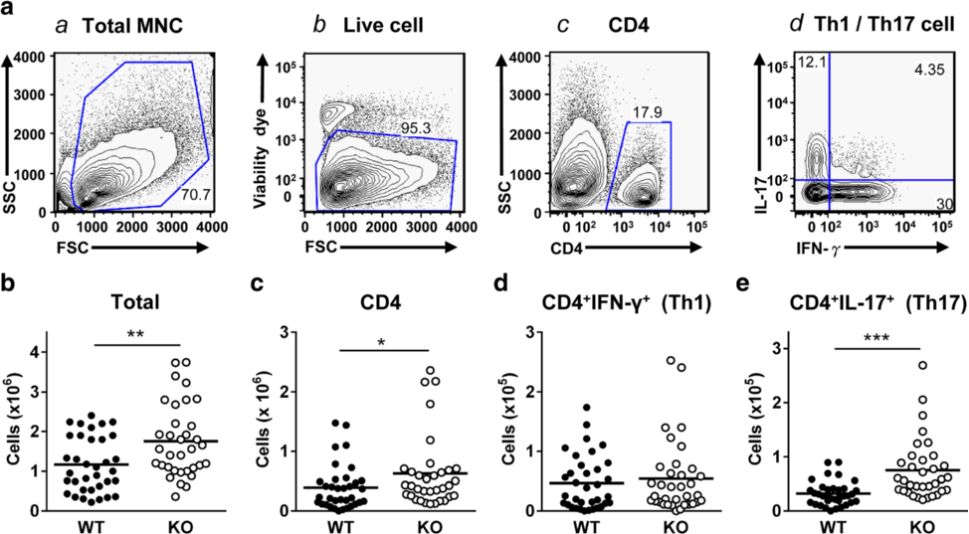
CNS-infiltrating Th17 cells are significantly elevated in CXCR3^−/−^ mice compared with WT mice. Mononuclear cells were isolated from the spinal cords of MOG-immunized WT (*n* = 35) and CXCR3^−/−^ (*n* = 34) mice at the peak of disease. Total and subpopulations of CNS-infiltrating lymphocytes were determined by surface staining and intracellular staining using flow cytometry as described in the “Methods” section. **a** The gating strategy is shown: *a* mononuclear cells from spinal cords were gated; *b* the live mononuclear cells were further gated from total mononuclear cells shown in *a*; *c* CD4^+^ T cells were further gated from live mononuclear cells shown in *b*; and *d* the subpopulation of CD4^+^IFN-γ^+^ T cells and CD4^+^IL-17^+^ T cells were identified from CD4^+^ T cells shown in *c*. **b** Total CNS-infiltrating leukocytes. **c** CNS-infiltrating CD4^+^ T cells. **d** CNS-infiltrating CD4^+^IFN-γ^+^ T cells. **e** CNS-infiltrating CD4^+^IL-17^+^ T cells. Each mouse is represented by a symbol. Data shown are from six independent experiments. *P < 0.05; **P < 0.01; ***P < 0.001

Given that EAE is a T cell-mediated inflammatory disease, we examined the subsets of infiltrating CD4^+^ effector T cells in the spinal cord at the peak of disease (day 15). Mononuclear cells were isolated from the spinal cords on day 15 post-immunization and immunostained for surface CD4 and intracellular IL-17 and IFN-γ by FACS. The gating strategy of FACS analysis is shown in Fig. 2a. Total CNS-infiltrating leukocytes were significantly increased in CXCR3^−/−^ mice compared with WT mice (*P* < 0.01, Fig. 2b), as were the total numbers of all CD4^+^ T cells infiltrating the spinal cord (*P* < 0.05, Fig. 2c). The increase in infiltrating CD4^+^ cells was further analyzed based on subpopulations. Infiltrating CD4^+^IFN-γ^+^ (Th1) cells were comparable between CXCR3^−/−^ mice and WT mice (Fig. 2d), whereas infiltrating CD4^+^IL-17^+^ (Th17) cells were significantly increased in CXCR3^−/−^ mice compared with WT mice (*P* < 0.001, Fig. 2e). The immunofluorescence staining of Th17 cells in frozen tissue sections of the spinal cord is also performed and shown in Additional file 2: Figure S2.

### CXCR3^−/−^ mice adoptively transferred with Th17 cells polarized from CD4^+^ T cells of MOG-immunized CXCR3^−/−^ mice are significantly more susceptible to EAE

**Fig. 3:**
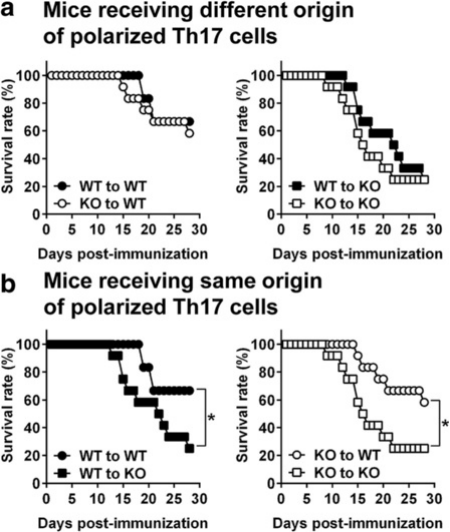
A CXCR3-deficient CNS milieu is responsible for the higher susceptibility of CXCR3^−/−^ mice to EAE. Splenocytes isolated from MOG-immunized WT or CXCR3^−/−^ mice on day 10 post-immunization were cultured under Th17-polarizing conditions in the presence of MOG_,_ IL-6, IL-23, and TGF-β for 72 h. Cultured CD4^+^ T cells were purified by MACS-positive selection, and 3 × 10^5^ CD4^+^IL-17^+^ cells were transferred into irradiated (4 Gy) WT or CXCR3^−/−^ mice followed by immunization with a suboptimal dose of MOG/CFA (10 μg/50 μg) and injection of PTX (200 ng/ml; day 0 and day 2). Four adoptive-transfer EAE experiments were performed: (1) WT (Th17) → WT, (2) WT (Th17) → CXCR3^−/−^, (3) CXCR3^−/−^ (Th17) → WT, and (4) CXCR3^−/−^ (Th17) → CXCR3^−/−^. Survival rates of recipient WT and CXCR3^−/−^ mice at various times post-immunization are shown. **a** Comparison of transfer of polarized Th17 cells with different origins into WT recipient mice (WT → WT vs. CXCR3^-/-^ → WT, *left panel*) or CXCR3^−/−^ recipient mice (WT → CXCR3^−/−^ vs. CXCR3^-/-^ → CXCR3^−/−^, *right panel*). **b** Comparison of transfer of polarized Th17 cells with the same origins into WT or CXCR3^−/−^ recipient mice (WT → WT vs. WT → CXCR3^−/−^, *left panel*; CXCR3^−/−^ → WT vs. CXCR3^−/−^ → CXCR3^−/−^, *right panel*). Data shown are from two independent experiments (12 mice for each adoptive-transfer EAE group). *P < 0.05; **P < 0.01; ***P < 0.001

Having established that infiltrating Th17 cells in the CNS are increased in CXCR3^−/−^ mice compared with WT mice during EAE (Fig. 2), we next addressed whether Th17 cells polarized from CXCR3^−/−^ CD4^+^ T cells were more detrimental for EAE development by performing adoptive-transfer EAE experiments. Donor WT or CXCR3^−/−^ mice were immunized with MOG, and splenocytes were collected at the subclinical phase (10 day post-immunization). Splenocytes were cultured with MOG under Th17-polarizing conditions followed by purification of CD4^+^ T cells. Polarized Th17 cells from WT or CXCR3^−/−^ mice were adoptively transferred into irradiated WT or CXCR3^−/−^ recipient mice followed by immunization with a suboptimal dose of MOG. WT recipient mice receiving Th17 cells polarized from CXCR3^−/−^ CD4^+^ T cells (CXCR3^−/−^ ➔ WT) showed a survival rate comparable to that of WT mice receiving Th17 cells polarized from WT CD4^+^ T cells (WT ➔ WT) (Fig. 3a, left panel). Similarly, CXCR3^-/-^ recipient mice receiving Th17 cells polarized from CXCR3^−/−^ CD4^+^ T cells (CXCR3^-/-^ ➔ CXCR3^−/−^) showed a survival rate comparable to that of CXCR3^-/-^ recipient mice receiving Th17 cells polarized from WT CD4^+^ T cells (WT ➔ CXCR3^−/−^) (Fig. 3a, right panel). These results suggest that Th17 cells polarized from WT or CXCR3^−/−^ CD4^+^ T cells may not be responsible for the severity of disease observed in CXCR3^−/−^ mice. However, we noted that survival rates were always low in cases where recipients were CXCR3^−/−^ mice (Fig. 3a, left panel vs. right panel). Applying another approach for comparing the survival rate between WT and CXCR3^−/−^ recipient mice, we found that irradiated CXCR3^−/−^ recipient mice adoptively transferred with Th17 cells polarized from WT or CXCR3^−/−^ CD4^+^ T cells always showed significantly increased morbidity compared with irradiated WT recipient mice receiving polarized Th17 cells from the same origin (*P* < 0.05; Fig. 3b). Because both WT and CXCR3^−/−^ mice received identical Th17 cells, the only difference that could account for the more severe EAE is the specific CNS microenvironment in the recipient mice. Taken together, these results demonstrate that Th17 cells polarized from either WT or CXCR3^−/−^ CD4^+^ T cells do not significantly contribute to the disease severity of CXCR3^−/−^ mice; instead, the CNS milieu in CXCR3^−/−^ mice is mainly responsible. On the basis of these results, we conclude that CXCR3 expressed on CNS-resident cells negatively regulates EAE pathogenesis.

### CXCR3^−/−^ recipient mice show increased CNS-infiltrating Th17 cells compared with WT recipient mice in adoptive-transfer EAE

**Fig. 4:**
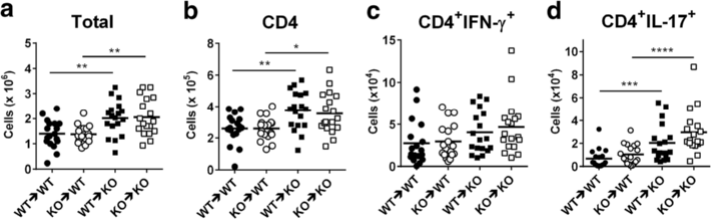
CXCR3^−/−^ recipient mice show increased CNS-infiltrating Th17 cells. Adoptive-transfer EAE was performed as described in Fig. 3, except that 2 × 10^5^ donor CD4^+^IL-17^+^ cells were used for adoptive-transfer experiments (WT → WT and WT → CXCR3^−/−^, 18 recipient mice for each group; CXCR3^−/−^ → WT and CXCR3^-/-^ → CXCR3^−/−^, 19 recipient mice for each group). Spinal cords were collected from recipient mice on day 12 post-immunization, and CNS-infiltrating cells were isolated. Different subsets of T cells were identified by surface and intracellular staining using flow cytometry. **a** Total CNS-infiltrating leukocytes. **b** CNS-infiltrating CD4^+^ T cells. **c** CNS-infiltrating CD4^+^IFN-γ^+^ T cells. **d** CNS-infiltrating CD4^+^IL-17^+^ T cells. Each mouse is represented by a symbol. Data shown are from three independent experiments. *P < 0.05; **P < 0.01; ***P < 0.001; ****P < 0.0001. The gating strategy for the FACS analysis of this figure is shown in Additional file 3: Figure S3

Given that CXCR3^−/−^ mice show more severe EAE and significantly increased infiltrating Th17 cells (Fig. 2), and that disease severity in CXCR3^−/−^ mice is associated with the CNS milieu (Fig. 3), we next investigated whether the CNS milieu in CXCR3^−/−^ was favorable for CNS-infiltrating Th17 cells. To this end, we performed adoptive-transfer EAE experiments in which Th17 cells polarized from WT or CXCR3^−/−^ mice were adoptively transferred into irradiated WT or CXCR3^−/−^ recipient mice followed by immunization with a suboptimal dose of MOG. Following transfer of Th17 cells of the same origin polarized either from WT or CXCR3^−/−^ CD4^+^ T cells, CXCR3^−/−^ recipient mice showed a significant increase in total CNS-infiltrating leukocytes (*P* < 0.01 for WT ➔ WT vs. WT ➔ CXCR3^−/−^ and CXCR3^−/−^ ➔ WT vs. CXCR3^−/−^ ➔ CXCR3^−/−^; Fig. 4a) and CD4^+^ T cells (*P* < 0.01 for WT ➔ WT vs. WT ➔ CXCR3^−/−^ and *P* < 0.05 for CXCR3^−/−^ ➔ WT vs. CXCR3^-/-^ ➔ CXCR3^−/−^; Fig. 4b) compared with WT recipient mice. Furthermore, the total number of CNS-infiltrating Th1 cells was comparable (Fig. 4c), whereas Th17 cells were significantly elevated in CXCR3^−/−^ recipient mice compared with WT recipient mice (*P* < 0.001 for WT ➔ WT vs. WT ➔ CXCR3^−/−^ and *P* < 0.0001 for CXCR3^−/−^ ➔ WT vs. CXCR3^-/-^ ➔ CXCR3^−/−^; Fig. 4d). These results show that a CXCR3-deficient CNS milieu indeed promotes better expansion of Th17 cells, regardless of their origin, than a WT CNS milieu.

Although WT or CXCR3^−/−^ mice receiving Th17 cells polarized from CXCR3^-/-^CD4^+^ T cells showed a tendency toward an increased number of CNS-infiltrating Th17 cells compared with mice that received Th17 cells polarized from WT CD4^+^ T cells (CXCR3^−/−^ ➔ WT vs. WT ➔ WT and CXCR3^−/−^ ➔ CXCR3^−/−^ vs. WT ➔ CXCR3^−/−^), this difference did not reach statistical difference (Fig. 4d). Notably, the rank order of increased CNS-infiltrating Th17 cells in adoptive-transfer EAE was CXCR3^−/−^ ➔ CXCR3^−/−^ **>** WT ➔ CXCR3^−/−^ **>** CXCR3^−/−^ ➔ WT **>** WT ➔ WT, further supporting the conclusion that a CXCR3-deficient CNS milieu has a greater impact on Th17 expansion during EAE than Th17 cells derived from CXCR3-deficient CD4^+^ T cells. The gating strategy for FACS analysis of total lymphocytes, CD4^+^ T cells, CD4^+^IFN-γ^+^, and CD4^+^IL-17^+^ is shown in Additional file 3: Figure S3

### Th17 cells infiltrating the spinal cord of CXCR3^−/−^ mice show increased proliferation compared to those infiltrating the spinal cord of WT mice

**Fig. 5:**
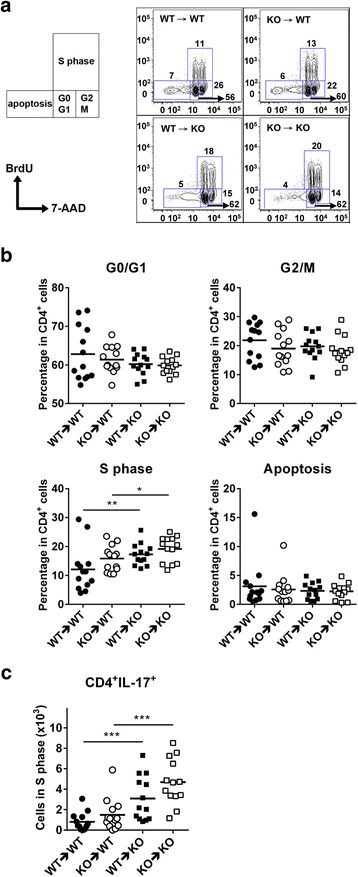
The CNS milieu of CXCR3^−/−^ mice promotes the proliferation of Th17 cells. Adoptive-transfer EAE was performed as described in Fig. 4. Recipient mice were injected with BrdU on day 11 post-immunization. At 24-h post-injection, the spinal cord was collected and CNS-infiltrating cells were isolated. The cells were immunostained with anti-CD4-APC-Cy7 followed by cell cycle analysis by quantification of cell-incorporated BrdU and total DNA content (7-AAD). **a** Representative flow cytometry plots for cell cycle analysis. **b** The percentage of CD4^+^ T cells in the apoptotic phase, G0/G1 phase, S phase, and G2/M phase of the cell cycle among the four groups. **c** The number of IL-17^+^ cells in CD4^+^BrdU^+^ (S phase) are shown for the four groups. Each group has 13 mice (*n* = 13). Each mouse is represented by a symbol. Data shown are from two independent experiments. **P < 0.01; ***P < 0.001

Since adoptive-transfer EAE experiments showed that the number of Th17 cells is significantly increased in the CXCR3-deficient CNS milieu compared with the WT CNS milieu (Fig. 4), we hypothesized that Th17 cells would proliferate better in a CXCR3-deficient CNS milieu. To test this hypothesis, we performed a cell cycle analysis of infiltrating CD4^+^ T cells in the spinal cord in the aforementioned adoptive-transfer EAE experiments (Fig. 4) by injecting recipient mice with BrdU and harvesting CNS-infiltrating cells 24 h later. The CNS-infiltrating cells were subjected to cell cycle analysis by immunostaining with fluorochrome-conjugated anti-BrdU and 7-ADD. As shown in Fig. 5a, a significantly higher percentage of WT CD4^+^ T cells were in S phase when adoptively transferred into CXCR3^−/−^ recipient mice than into WT recipient mice (*P* < 0.01 for WT ➔ WT vs. WT ➔ CXCR3^−/−^; Fig. 5b). Similarly, CXCR3^−/−^ CD4^+^ T cells showed a significantly higher percentage of cells in S phase when adoptively transferred into CXCR3^−/−^ recipient mice than into WT recipient mice (*P* < 0.05 for CXCR3^−/−^ ➔ WT vs. CXCR3^−/−^ ➔ CXCR3^−/−^; Fig. 5b). No significant difference was observed in the percentages of cells in G0/G1, G2/M, or apoptosis phases among these four groups of recipient mice (Fig. 5b). We further analyzed the number of CD4^+^IL-17^+^ T cells in S phase. As expected, the number of CD4^+^ IL-17^+^ T cells in S phase was significantly increased in CXCR3^−/−^ recipient mice compared with WT recipient mice (*P* < 0.001 for WT ➔ WT vs. WT ➔ CXCR3^−/−^ and CXCR3^−/−^ ➔ WT vs. CXCR3^−/−^ ➔ CXCR3^−/−^; Fig. 5c). The rank order of the increase in CNS-infiltrating Th17 cells in adoptive-transfer EAE experiments (CXCR3^−/−^ ➔ CXCR3^−/−^ **>** WT ➔ CXCR3^−/−^ **>** CXCR3^−/−^ ➔ WT **>** WT ➔ WT; Fig. 4d) is consistent with the rank order of CD4^+^IL-17^+^ T cell proliferation (CXCR3^-/-^ ➔ CXCR3^−/−^ **>** WT ➔ CXCR3^−/−^ **>** CXCR3^−/−^ ➔ WT **>** WT ➔ WT; Fig. 5c). These results suggest that the CXCR3-deficient CNS milieu has a greater impact on the increase in CNS-infiltrating Th17 cells observed in CXCR3^−/−^ mice than does the increased proliferative capability of Th17 cells polarized from CXCR3-deficient CD4^+^ T cells. The gating strategy for FACS analysis of cell cycle phase of CD4^+^ T cells is shown in Additional file 4: Figure S4.

The results from Figs. 4 and 5 show that the increase in CNS-infiltrating Th17 cells in CXCR3^−/−^ mice during EAE is likely attributable to the extrinsic effect of a pro-Th17 milieu generated by the CXCR3-deficient CNS. Notably, although CXCR3 mainly regulates effector lymphocyte trafficking to inflamed tissues, our current study provides the first demonstration that a CXCR3 deficiency in the CNS has a greater impact on the pathogenesis of EAE.

### Cytokines required for Th17 expansion and recruitment are significantly increased in the spinal cord in CXCR3^−/−^ mice compared with WT mice

**Fig. 6:**
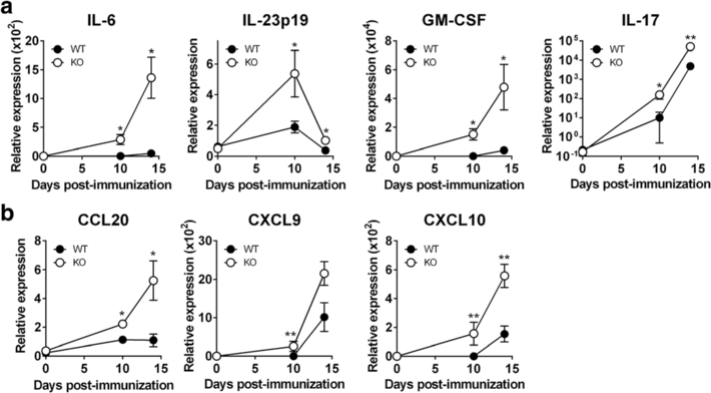
mRNAs for inflammatory chemokines and cytokines required for Th17 cell expansion are increased in CXCR3^−/−^ mice during EAE. WT (*n* = 5) and CXCR3^−/−^ (*n* = 5) mice were immunized with MOG. Spinal cords were collected on days 0, 10, and 14 post-immunization, and total RNA was extracted and subjected to real-time PCR analysis to determine the mRNA levels for cytokines (**a**) and chemokines (**b**). mRNA levels of target genes, normalized to the level of *GAPDH*, were analyzed using the ΔΔCt method. *n* stands for the number of mice. *P < 0.05; **P < 0.01

Having found that a CXCR3-deficient CNS milieu promotes Th17 cell expansion (Figs. 4 and 5), we next examined whether a CXCR3 deficiency generates a CNS microenvironment preferential for Th17 proliferation, survival, maintenance, and recruitment. An analysis of cytokine gene expression in the spinal cord from MOG-immunized WT and CXCR3^−/−^ mice on days 0, 10 (preclinical phase), and 14 (peak phase) revealed that mRNAs for cytokines required for the expansion of Th17 cells were significantly increased in the spinal cord of CXCR3^−/−^ mice compared with WT mice at both pre-clinical (day 10) and peak (day 14) phases (Fig. 6). The expression levels of cytokine genes *IL-6*, *IL-23p19*, *GM-CSF*, and *IL-17* responsible for the expansion of Th17 cells (Fig. 6a) and chemokine gene *CCL20* (Fig. 6b) responsible for the recruitment of Th17 cells into inflammatory sites were significantly elevated in the spinal cord of CXCR3^−/−^ mice. The levels of mRNAs for the CXCR3 ligands *CXCL9* and *CXCL10* were also significantly increased (Fig. 6b).

### CXCR3-deficient glial cells stimulated with LPS significantly increase IL-23 and CCL20 secretion

**Fig. 7:**
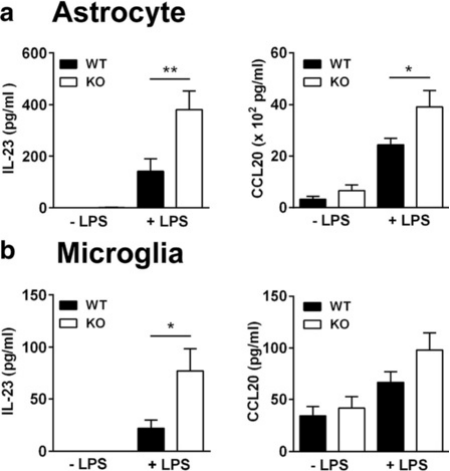
IL-23 and CCL20 production are significantly increased in glial cells derived from the cortex of neonatal CXCR3^−/−^ mouse brains compared with those from WT mice. The brain cortex was collected from neonatal mice (day 1), and cells were disaggregated. Disaggregated cells were cultured in glial cell enrichment medium for 12 days followed by the separation of astrocytes (**a**) from microglia (**b**), as described in the “Methods” section. Astrocytes and microglia were stimulated with LPS for 24 h, and IL-23p19 and CCL20 levels in collected culture supernatants were measured by ELISA. Data represent means ± SEM from eight independent experiments with three neonatal mice for each experiment. *P < 0.05; **P < 0.01

Given that production of pro-Th17 cytokines is increased in the CXCR3-deficient spinal cord (Fig. 6) and that glial cells, astrocytes and microglia, in CNS parenchymal tissues express CXCR3 [26, 28, 36], we further examined whether glial cells lacking CXCR3 preferentially produced cytokines required for Th17 cell generation and recruitment during inflammation. Primary glial cells were isolated from the cortex of neonatal mouse brains and differentiated into microglia and astrocytes. Microglia and astrocytes were stimulated with lipopolysaccharide (LPS) followed by an analysis of the secretion of IL-23 and CCL20, which are required for the expansion and recruitment of Th17 cells, respectively. As shown in Fig. 7, both astrocytes (Fig. 7a) and microglia (Fig. 7b) differentiated from the CXCR3^−/−^ brain cortex showed increased levels of IL-23 and CCL20 compared with those differentiated from WT mice. Primary glial cells isolated from CXCR3^−/−^ mice produced greater amounts of cytokines compared with those isolated from WT mice (Fig. 7), suggesting an intrinsic increase in pro-Th17 cytokines in CXCR3-deficient glial cells.

### CXCR3^−/−^ mice exhibit a significant increase in the number of glial cells in the spinal cord compared with WT mice during EAE

**Fig. 8:**
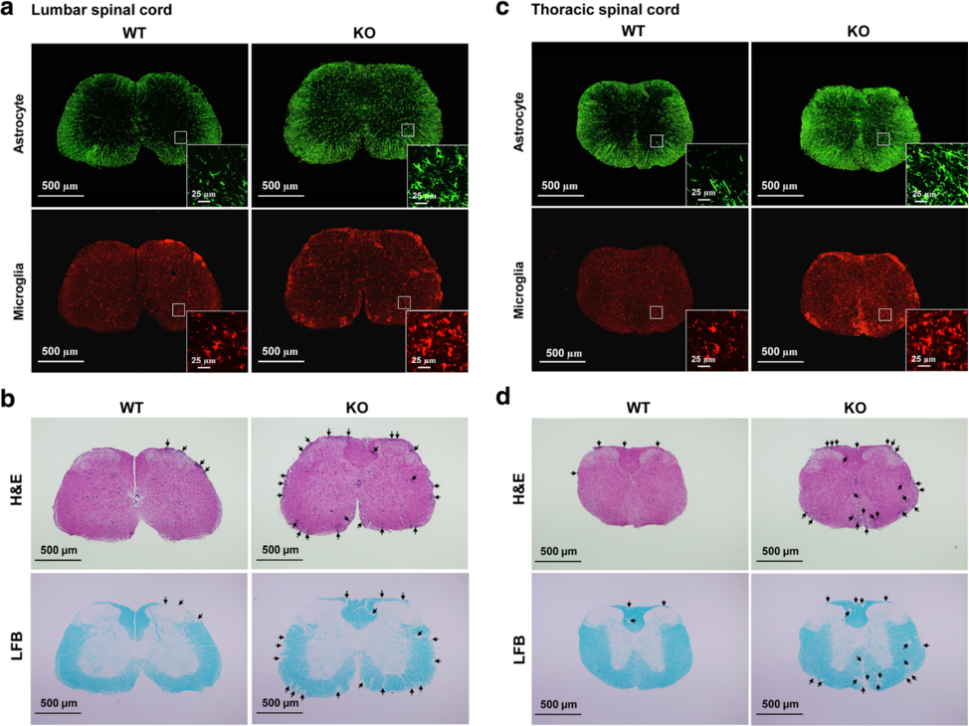
CXCR3^−/−^ mice show elevated numbers of glial cells and increased leukocyte infiltration and demyelination in the spinal cord during EAE. EAE was induced in WT and CXCR3^−/−^ mice as described in Fig. 1. At 15-days post-immunization, lumbar (**a**, **b**) and thoracic (**c**, **d**) spinal cords were collected and subjected to immunofluorescence staining (**a**, **c**) and H&E as well as LFB staining (**b**, **d**). Immunofluorescence staining for Iba1 (microglial cells) and GFAP (astrocytes) were performed as described in the “Methods” section. *Magnified insets* highlight the morphology of activated glial cells in lumbar spinal cords (**a**) or in thoracic spinal cords (**c**). Infiltrating leukocytes (**b**, **d**; *upper panel*) and demyelination (**b**, **d**; *lower panel*) were detected by H&E staining and LFB staining, respectively, in lumbar (**b**) and thoracic (**d**) spinal cords. *Arrows* indicate infiltrating leukocytes (**b**, **d**; *upper panel*) and demyelination (**b**, **d**; *lower panel*). Data are representative of three independent experiments

The levels of cytokines that favor Th17 cell expansion and recruitment were significantly increased in the spinal cord of CXCR3^−/−^ mice compared with WT mice (Fig. 6). This increase could result from enhanced cytokine production by CXCR3-deficient glial cells and/or an increase in the number of glial cells in the spinal cord of CXCR3^−/−^ mice during EAE. The pro-Th17 cytokine production in CXCR3-deficient glial cells was significantly increased compared to WT glial cells, likely owing to an intrinsic effect (Fig. 7). We next examined whether an increase in the number of glial cells in the spinal cord of CXCR3^−/−^ mice also contributed to the increased cytokine expression in CXCR3^−/−^ mice during EAE. Spinal cord sections from lumbar (Fig. 8a, b) and thoracic regions (Fig. 8c, d) of WT and CXCR3^−/−^ mice were subjected to immunofluorescence staining. Astrocytes and microglia were detected using antibodies to GFAP and Iba1, respectively. Astrocytes were not evident within gray matter in naïve WT or CXCR3^−/−^ mice (data not shown). However, the number of astrocytes (GFAP^+^) within gray matter was significantly increased during EAE in both WT and CXCR3^−/−^ mice, but this increase was greater in CXCR3^−/−^ mice (Fig. 8a, c). Notably, astrocytes within white matter in CXCR3^−/−^ mice became intensively GFAP immunoreactive (Fig. 8a, c), indicating more severe inflammation in CXCR3^−/−^ mice compared with WT mice. These results suggest that astroglial proliferation and hypertrophy in the spinal cord is significantly greater in CXCR3^−/−^ mice than in WT mice. Similarly, microglia within white matter were significantly increased in the spinal cord of CXCR3^−/−^ mice compared with WT mice (Fig. 8a, c). The increased number of glial cells in the spinal cord of CXCR3^−/−^ mice may also contribute to the increased levels of pro-Th17 cytokines/chemokines, leading to an increase in CNS-infiltrating Th17 cells and thus to the increased EAE severity in CXCR3^−/−^ mice. Notably, the increased number of glial cells in CXCR3^−/−^ mice reflected the severity of the inflammation, as evidenced by increased leukocyte infiltration (Fig. 8b, d, upper panel) and demyelination (Fig. 8, d, lower panel) in the spinal cord. Taken together, these results indicate that the increased pro-Th17 cytokine production observed in the spinal cord of CXCR3^−/−^ mice during EAE results from both the increased number of glial cells in spinal cords (Fig. 8) and the increased pro-Th17 cytokine production per glial cell (Fig. 7).

### CXCR3 is expressed on glial cells and attenuates NF-κB activation in glioblastoma cells

**Fig. 9:**
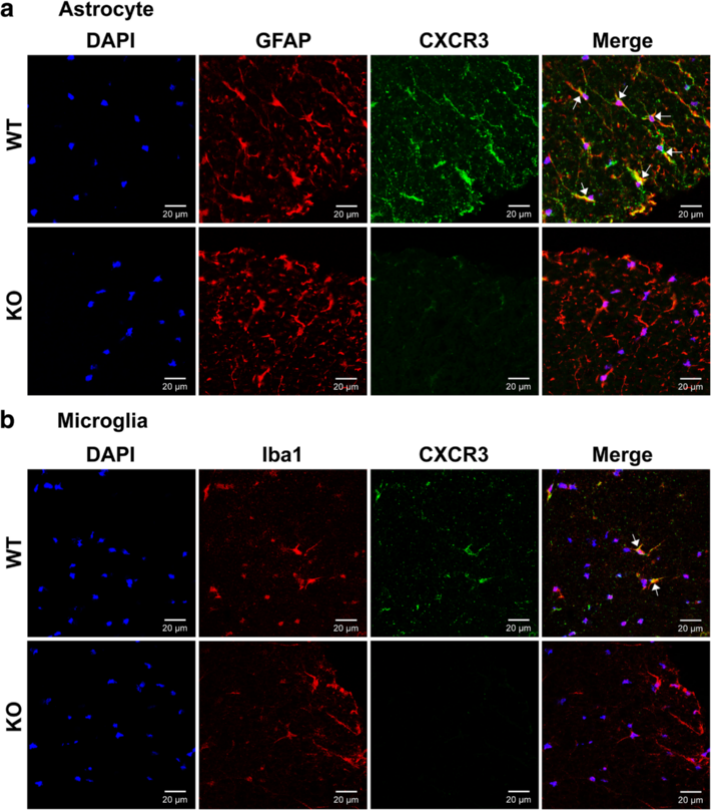
CXCR3 is expressed on both astrocytes and microglia. Frozen sections of lumbar spinal cord collected from WT and CXCR3^−/−^ mice at the peak of disease (day 15) were subjected to immunofluorescence staining as described in the “Methods” section. **a** Double-labeling fluorescent staining shows CXCR3 on astrocytes. Anti-CXCR3 (*green*), anti-GFAP (*red*), DAPI (*blue*). **b** Double-labeling fluorescent staining shows CXCR3 on microglial. Anti-CXCR3 (*green*), anti-Iba1 (*red*), DAPI (*blue*). The *arrows indicate* co-localization of CXCR3 and glial cell markers. The immunofluorescence staining with isotype control antibody for anti-CXCR3 is shown in Additional file 5: Figure S5. Data are representative of three independent experiments

**Fig. 10:**
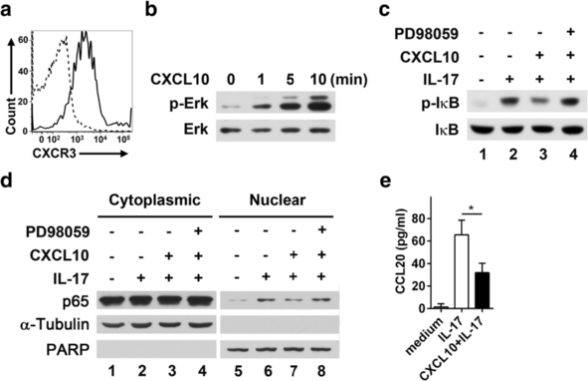
CXCR3 signaling negatively regulates NF-κB activation induced by IL-17 in a glioblastoma cell line. **a** U87MG cells were stained with an anti-CXCR3-APC (*solid line*) or isotype control (*dashed line*) antibody followed by FACS analysis. **b** U87MG cells were serum-starved for 6 h followed by stimulation with CXCL10 (100 ng/ml). Proteins in whole-cell lysates, prepared at the indicated times, were resolved by SDS-PAGE followed by immunoblotting with anti-p-ERK and anti-ERK antibodies. **c** U87MG cells were serum-starved for 6 h and preincubated with or without PD98059 (10 ng/ml) for 15 min followed by incubation with CXCL10 (100 ng/ml) for 60 min. Cells were then stimulated with IL-17 (100 ng/ml) for 15 min followed by cell lysis. Whole-cell lysates were subjected to SDS-PAGE followed by immunoblotting with antibodies to IκB and phospho-IκB. **d** Similar to **b** except that cells were stimulated with IL-17 for 30 min and cell lysates were fractionated into cytoplasmic and nuclear fractions. Proteins in whole-cell lysates, cytoplasmic fractions, and nuclear fractions were resolved by SDS-PAGE followed by immunoblotting with antibodies to NF-κB p65, *α*-tubulin, and PARP. PARP and *α*-tubulin were used as markers for the nuclear and the cytoplasmic fractions, respectively. **e** U87MG cells were cultured in complete medium containing IL-17 (100 ng/ml) or IL-17 (100 ng/ml) plus CXCL10 (100 ng/ml) for 48 h, after which CCL20 in culture supernatants was detected by ELISA. Data represent means ± SEM from four independent experiments. *P < 0.05

Our data showed that CXCR3-deficient glial cells produce increased amounts of cytokines/chemokines compared with WT glial cells (Figs. 6 and 7). We hypothesized that CXCR3 signaling in glial cells might negatively regulate cytokine production. If this was the case, the absence of CXCR3 on glial cells would be predicted to amplify cytokine production. We first examined the expression of CXCR3 on glial cells of the spinal cord during peak of EAE by immunofluorescence staining. Comparing to isotype control antibody for anti-CXCR3 shown in Additional file 5: Figure S5, we found that CXCR3 expression was detected on both astrocytes (Fig. 9a) and microglia (Fig. 9b), consisting with previous reports that glial cells express CXCR3 [25–28]. We then investigated whether CXCR3 signaling in glial cells was able to regulate cytokine production negatively using an in vitro experiment with a glioblastoma cell line, U87MG, which expresses CXCR3 (Fig. 10a). It has been shown that CXCR3 signaling induces ERK activation [37, 38], that activated ERK attenuates NF-κB activation [39–41], and that activated NF-κB is required for the transcriptional regulation of *IL-23p19* and *CCL20* [42–45]. We hypothesized that CXCR3 signaling might reduce production of IL-23 and CCL20 in glial cells through such a mechanism. Stimulation of U87MG cells with the CXCR3 ligand CXCL10 induced ERK phosphorylation (Fig. 10b), whereas stimulation with IL-17 significantly induced IκB phosphorylation (Fig. 10c, lane 1 vs. lane 2). However, IL-17-induced IκB phosphorylation was significantly reduced in cells pretreated with CXCL10 (Fig. 10c, lane 2 vs. lane 3). Similarly, the nuclear translocation of NF-κB induced by IL-17 stimulation was also significantly reduced in cells pretreated with CXCL10 (Fig. 10d, lane 6 vs. lane 7). Furthermore, pretreatment of cells with PD98059, an ERK inhibitor, completely prevented CXCR3 signaling from attenuating IL-17-induced IκB phosphorylation (Fig. 10c, lane 3 vs. lane 4) and NF-κB activation (Fig. 10d, lane 7 vs. lane 8). To show that attenuated NF-κB activation mediated by CXCR3-signaling-induced ERK was indeed able to reduce CCL20 production, we measured CCL20 secretion in the supernatants of cultured U87MG cells. As expected, the CCL20 secretion induced by IL-17-stimulated U87MG cells was also significantly reduced in the presence of CXCL10 (Fig. 10e). These results further confirm that the ERK activation elicited by CXCR3 signaling indeed attenuates NF-κB activation, leading to a subsequent decrease in CCL20 production. Collectively, these results suggest that CXCR3-expressing glial cells play a role in negatively regulating pro-Th17 cytokine/chemokine production by suppressing NF-κB activation.

## Discussion

Using both active EAE and adoptive-transfer EAE models, we have demonstrated that CXCR3^−/−^ mice exhibit increased infiltrating Th17 cells in the spinal cord, leading to the development of more severe EAE. We have further demonstrated that the increase in CNS-infiltrating Th17 cells in CXCR3^-/-^ mice is attributable to the increased number of activated glial cells in the CNS as well as increased levels of pro-Th17 cytokines preferentially produced by CXCR3-deficient glial cells. Moreover, we identified suppression of NF-κB activation by CXCR3 signaling in glial cells as a possible mechanism underlying the negative regulation of pro-Th17 cytokines. Notably, our findings demonstrate a critical role of CXCR3 expressed on glial cells, showing that it prevents the overproduction of Th17 cells by modifying the CNS parenchyma microenvironment so as to limit the expansion and recruitment of Th17 cells during EAE, thus ameliorating disease severity.

CXCR3 and its ligands (CXCL9, CXCL10, CXCL11) are thought to govern the recruitment of leukocytes to the sites of inflammation and infection [15, 46]. One would expect that a deficiency of CXCR3 would reduce the recruitment of effector lymphocytes to the sites of inflammation during EAE, thus conferring resistance to EAE. On the contrary, two previous studies [29, 30], together with our current findings, point to CXCR3^−/−^ mice being more susceptible to EAE than WT mice, suggesting that CXCR3 signaling plays a protective role rather than a detrimental role in EAE. Interestingly, although these two previous studies and our study all show the susceptibility of CXCR3^−/−^ mice to EAE [29, 30], each has proposed a distinct mechanism. It is known that Th17 cells are crucial for EAE pathogenesis [9–12]. Consistent with this notion, we found a significant increase in CNS-infiltrating Th17 cells, but not Th1 cells, in CXCR3^−/−^ mice compared with WT mice (Figs. 2 and 4). Given that ligand-bound CXCR3 promotes IFN-γ production [47] and that the Th1-associated cytokines IFN-γ and IL-12 are able to antagonize Th17 cell differentiation and inhibit IL-23-driven proliferation of Th17 cells [48, 49], CXCR3^−/−^ mice would be expected to have a lower number of Th1 cells and thus fail to efficiently antagonize the proliferation of Th17 cells, leading to an increase in infiltrating Th17 cells in the spinal cord. Interestingly, we did not find a reduction in the number of Th1 cells in CXCR3^−/−^ mice compared with WT mice; therefore, the increase in CNS-infiltrating Th17 cells in CXCR3^−/−^ mice cannot be explained by a reduced number of CNS-infiltrating Th1 cells. Of particular interest, our study demonstrates that the increase in CNS-infiltrating Th17 cells in CXCR3^−/−^ mice results from an increase in the number of CNS glial cells, which preferentially generate a cytokine milieu favorable for Th17 cell expansion. Our study is the first to demonstrate the importance of CXCR3 signaling in glial cells during EAE, establishing that CXCR3 expressed on glial cells negatively regulates Th17 cell infiltration into the CNS.

Conflicting results regarding the role of CXCR3 and its ligands in EAE have been reported. Our studies along with others have demonstrated that CXCR3^−/−^ mice are susceptible to EAE [29, 30]. However, results showing resistance to EAE in CXCR3-deficiency mice, or in mice in which CXCR3 was neutralized, have been reported [31–34]. These discrepancies might be due to the species or strain of mice used (rat or B57BL/6 or SJL/J), to the antigens used for immunization (CNS homogenate, MOG, myelin basic protein, or myelin proteolipid protein), to the model used (acute vs. chronic, pharmacologic antagonism, antibody), and to differences in microbiota (co-housing mice, littermate control). Therefore, caution should be exercised in interpreting the data on the role for CXCR3 in EAE; since depending on the model used, CXCR3 signaling may have either pathogenic or regulatory effects. The disparate results from the studies of CXCR3 in EAE are summarized in Additional file 6: Table S1. Studies of the roles for CXCR3 in EAE have been focused on the leukocyte recruitment. However, our study is the first to demonstrate the functional importance of CXCR3 on glial cells negatively regulating Th17 cell expansion during EAE.

Migration of Th17 cells to the CNS has been linked to the induction of inflammation in MS [50] and EAE [9]. The CCR6-CCL20 axis is important for directing Th17 cell migration because Th17 cells preferentially express CCR6, whose ligand is CCL20 [51]. Two recent studies have investigated the mechanism by which Th17 cells enter and/or accumulate in the CNS from peripheral lymphoid organs [12, 52], proposing two different models. Reboldi et al. showed that Th17 cells enter the CNS through the choroid plexus, where CCL20 is highly expressed, and through a CCR6-CCL20-dependent mechanism and migrate into the subarachnoid space of the CNS; there, they are activated/proliferate, triggering the initial inflammation during EAE [12]. Arima et al. reported that the entry site for Th17 cells into the CNS during EAE is at dorsal vessels of the fifth lumber cord, also via the CCR6-CCL20 axis, reflecting the high expression of CCL20 in these vessels [52]. These two studies both implicate the importance of CCR6-CCL20 axis in the initial entry of Th17 cells into the CNS. We found that the levels of CCR6 in Th17 cells polarized from WT CD4^+^ T cells and CXCR3^−/−^ CD4^+^ T cells were comparable (data not shown); therefore, we anticipate that the initial entry of Th17 cells into the CNS would be comparable between WT and CXCR3^−/−^ mice. Following the initial trigger of Th17 cell entry, the infiltrating Th17 cells then further migrate into the CNS parenchyma, where they may undergo further expansion [12, 53, 54]. Given that the CXCR3-deficient spinal cord produces increased levels of cytokines and chemokines favorable for the expansion of Th17 cells compared to the WT spinal cord (Fig. 6) and that Th17 cells in CXCR3^−/−^ mice show better expansion than that those in WT mice (Figs. 4 and 5c), the CXCR3-deficient CNS parenchyma likely generates a favorable microenvironment for Th17 cell expansion. Based on the studies from Reboldi et al. [12] and from Arima et al. [52] together with our study, we propose that the increase in CNS-infiltrating Th17 cells observed in CXCR3^−/−^ mice during EAE is attributable to increased Th17 expansion in the CNS parenchyma rather than an increase in the initial entry of Th17 cells into the CNS. Furthermore, CNS-infiltrating Th17 cells are significantly increased in CXCR3^−/−^ mice during EAE, likely owing to the increased number of glial cells in the spinal cord of CXCR3^−/−^ mice (Fig. 8) and to the preferential production of pro-Th17 cytokines in CXCR3-deficient glial cells (Figs. 6 and 7).

Inhibition of NF-κB in astrocytes has been shown to reduce expression of cytokines and chemokines, resulting in decreased expansion and recruitment of effector leukocytes and subsequent amelioration of EAE [55–57]. NF-κB is an essential transcription factor for the induction of both IL-23 [42, 43] and CCL20 [45], which are important for Th17 expansion. Our data showed that CXCR3-deficient glial cells produce more IL-23 and CCL20 than WT glia cells (Fig. 7), suggesting the possibility that CXCR3 signaling negatively regulates pro-Th17 cytokine production in glial cells. The relevance of this pathway was confirmed by in vitro experiments showing that NF-κB activation was suppressed by activated ERK elicited by CXCR3 signaling. Consistent with these findings, it has been reported that inhibition of NF-κB in neuroectodermal cells ameliorates EAE [55, 57]. The detailed molecular mechanism by which CXCR3-mediated ERK activity attenuates NF-κB activation in glial cells warrants further investigation.

EAE and MS are complicated diseases involving both immunological and neurological dysfunctions. The current study provides evidence that CXCR3 in glial cells plays a protective role during EAE, demonstrating that, in addition to its well-known functions in immune cells, CXCR3 on CNS resident cells also plays an important physiological role during EAE.

## Conclusions

This study identifies a protective role of CXCR3 during EAE due to its activity on astrocytes and microglia, adding to the receptor’s well-known, more conventional immunologic functions. Our data demonstrate that CXCR3 signaling in glial cells attenuates neuroinflammation during EAE by limiting pro-Th17 cytokine production, thereby restraining Th17 cell expansion, reducing CNS-infiltrating Th17 cells, and subsequently ameliorating EAE. We further provide a possible underlying mechanism in which ERK activation elicited by CXCR3 signaling attenuates the activation of NF-κB required for the induction of pro-Th17 cytokines.

## Additional files

Additional file 1: Figure S1. CXCR3^-/-^ mice have increased mononuclear cells infiltrating in the spinal cord as compared with WT mice. The consecutive sections of the spinal cord (L1) were isolated from MOG-immunized WT and CXCR3^-/-^ (KO) mice at peak of disease (day 15). LFB staining was performed for detecting demyelinating. The consecutive sections of WT1 to WT12 and KO1 to KO12 were cut rostrally after the sections shown in Fig. 1b. Mononuclear cells infiltrating in white matter were shown as cresyl violet positive cells (purple, original magnification × 100). (PDF 1986 kb) Additional file 2: Figure S2. Th17 cells are detected in the spinal cord by immunofluorescence assay. **a** Frozen sections of the spinal cord from MOG-immunized WT and CXCR3^-/-^ (KO) mice at peak of disease (day 15) were subjected to immunofluorescence staining with rat anti-mouse CD4 / biotin conjugateddonkey anti-rat / Alexa 555-conjugated streptavidin (red) and goat anti-mouse IL-17 / Alexa 488-conjugated donkey anti-goat (green) followed by counterstaining with DAPI (blue). Arrowheads indicate IL-17^+^ cells and arrows indicate CD4^+^IL-17^+^ cells (Th17). **b** Same procedure in (a) was performed except that goat antimouse IL-17 was replaced by isotype control antibody. (PDF 375 kb) Additional file 3: Figure S3. The gating strategy of FACS analysis to identify subpopulations of CD4^+^IL-17^+^ and CD4^+^IFN-γ^+^ cells shown in Fig. 4. Mononuclear cells were isolated from the spinal cord of WT and CXCR3^-/-^ recipient mice at day 12 post-immunization and subjected to FACS analysis. Lymphocytes were gated according to forward and side scatter and then subsequently gated on CD4^+^ cells. CD4^+^ cells were further gated to analyze IFN-γ^+^ cells (CD4^+^IFN-γ^+^, Th1) and IL-17^+^ cells (CD4^+^IL-17^+^, Th17). Data are representative of three independent experiments. (PDF 226 kb) Additional file 4: Figure S4. The gating strategy of FACS analysis to identify proliferating Th17 cells shown in Fig. 5. Mononuclear cells were isolated from the spinal cord of MOG-immunized WT and CXCR3^-/-^ recipient mice at day12 post-immunization and subjected to FACS analysis. Lymphocytes were gated according to forward and side scatter and then subsequently gated on CD4^+^ cells. CD4^+^ cells were further gated to analyze cell cycle phase distribution. The phases of the cell cycle of CD4 cells were indicated (G0/G1: BrdU- 7-AAD^+^; G2/M: BrdU^-^ 7-AAD^high^; S: BrdU^+^ 7-AAD^high^; Apoptosis: BrdU^-^ 7-AAD^low^). CD4^+^ cells in S phase were further gated to analyze IL-17^+^ cells (CD4^+^IL-17^+^, Th17). Data are representative of two independent experiments. (PDF 183 kb) Additional file 5: Figure S5. Isotype control antibody for anti-CXCR3 in Fig. 9. Frozen sections were subjected to immunofluorescence staining as describe in Fig. 9 except that anti-CXCR3 was replaced with isotype control antibody. **a** Astrocytes were marked as GFAP^+^ cells. **b** Microglia were marked as Iba1^+^ cells. GFAP: red, Iba1: red, isotype control antibody for anti-CXCR3: green, DAPI: blue. (PDF 981 kb) Additional file 6: Table S1. The effect of CXCR3 in various studies. (DOCX 17 kb)

## Abbreviations

CNS: central nervous system
EAE: experimental autoimmune encephalomyelitis
ELISA: enzyme-linked immunosorbent assay
FACS: fluorescence-activated cell sorting
MOG: myelin oligodendrocyte glycoprotein
MS: multiple sclerosis
PTX: pertussis toxin
WT: wild-type
