# Supplementary material for: CXCR3 signaling in glial cells ameliorates experimental autoimmune encephalomyelitis by restraining the generation of a pro-Th17 cytokine milieu and reducing CNS-infiltrating Th17 cells
Source: J Neuroinflammation. 2016 Apr 11;13:76. doi: 10.1186/s12974-016-0536-4 (PMC4828793; doi:10.1186/s12974-016-0536-4)
Supplement: Additional file 1: Figure S1. — CXCR3-/- mice have increased mononuclear cells infiltrating in the spinal cord as compared with WT mice. The consecutive sections of the spinal cord (L1) were isolated from MOG-immunized WT and CXCR3-/- (KO) mice at peak of disease (day 15). LFB staining was performed for detecting demyelinating. The consecutive sections of WT1 to WT12 and KO1 to KO12 were cut rostrally after the sections shown in Fig. 1b. Mononuclear cells infiltrating in white matter were shown as cresyl violet positive cells (purple, original magnification × 100). (PDF 1986 kb) [file 12974_2016_536_MOESM1_ESM.pdf]

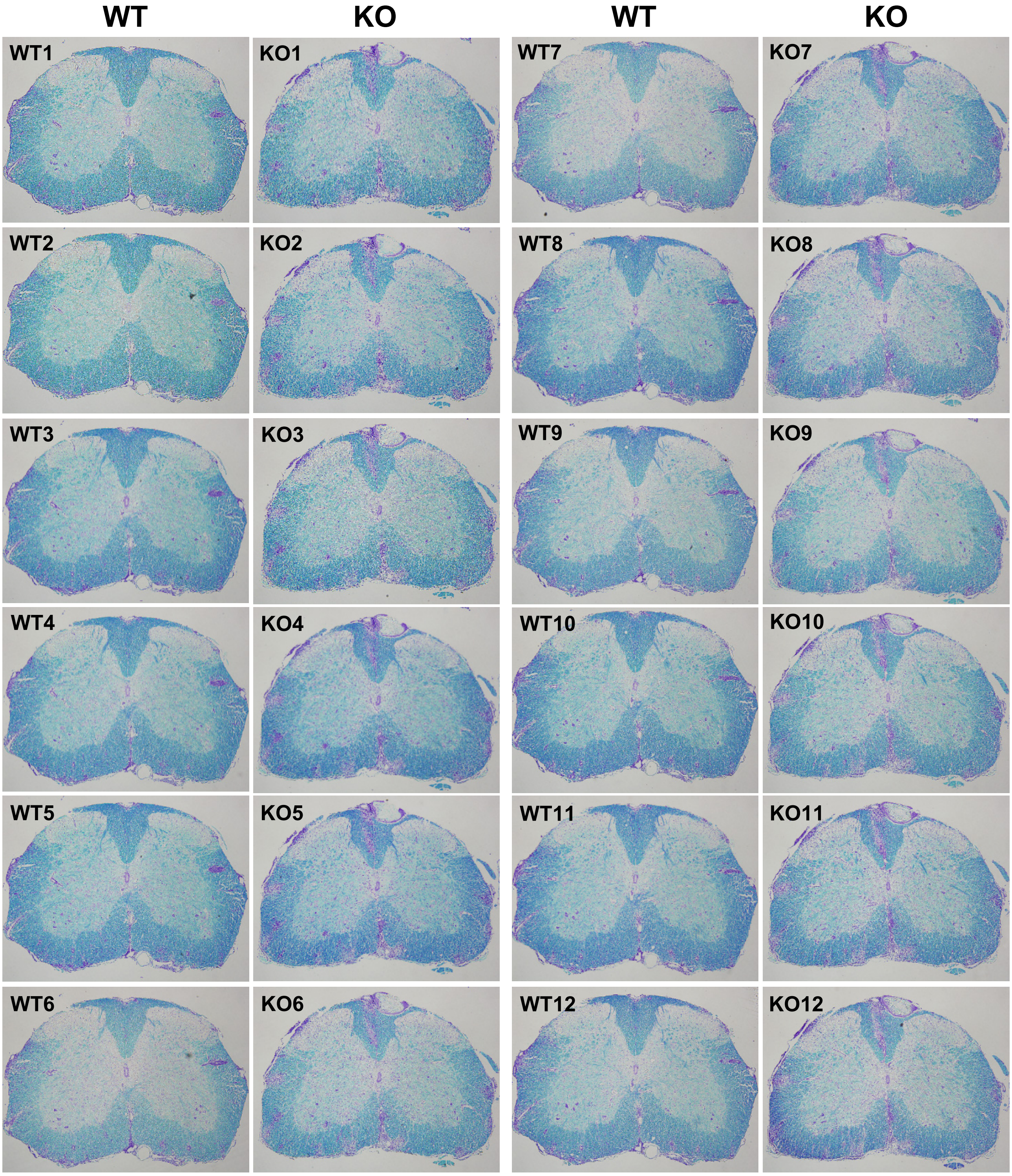

Figure S1. CXCR3$^{-/-}$ mice have increased mononuclear cells infiltrating in the spinal cord as compared with WT mice. The consecutive sections of the spinal cord (L1) were isolated from MOG-immunized WT and CXCR3$^{-/-}$ (KO) mice at peak of disease (day 15). LFB staining was performed for detecting demyelinating. The consecutive sections of WT1 to WT12 and KO1 to KO12 were cut rostrally after the sections shown in Figure 1B. Mononuclear cells infiltrating in white matter were shown as cresyl violet positive cells (purple, original magnification $\times$ 100).
