# Supplementary material for: CXCR3 signaling in glial cells ameliorates experimental autoimmune encephalomyelitis by restraining the generation of a pro-Th17 cytokine milieu and reducing CNS-infiltrating Th17 cells
Source: J Neuroinflammation. 2016 Apr 11;13:76. doi: 10.1186/s12974-016-0536-4 (PMC4828793; doi:10.1186/s12974-016-0536-4)
Supplement: Additional file 2: Figure S2. — Th17 cells are detected in the spinal cord by immunofluorescence assay. a Frozen sections of the spinal cord from MOG-immunized WT and CXCR3-/- (KO) mice at peak of disease (day 15) were subjected to immunofluorescence staining with rat anti-mouse CD4 / biotin conjugateddonkey anti-rat / Alexa 555-conjugated streptavidin (red) and goat anti-mouse IL-17 / Alexa 488-conjugated donkey anti-goat (green) followed by counterstaining with DAPI (blue). Arrowheads indicate IL-17+ cells and arrows indicate CD4+IL-17+ cells (Th17). b Same procedure in (a) was performed except that goat antimouse IL-17 was replaced by isotype control antibody. (PDF 375 kb) [file 12974_2016_536_MOESM2_ESM.pdf]

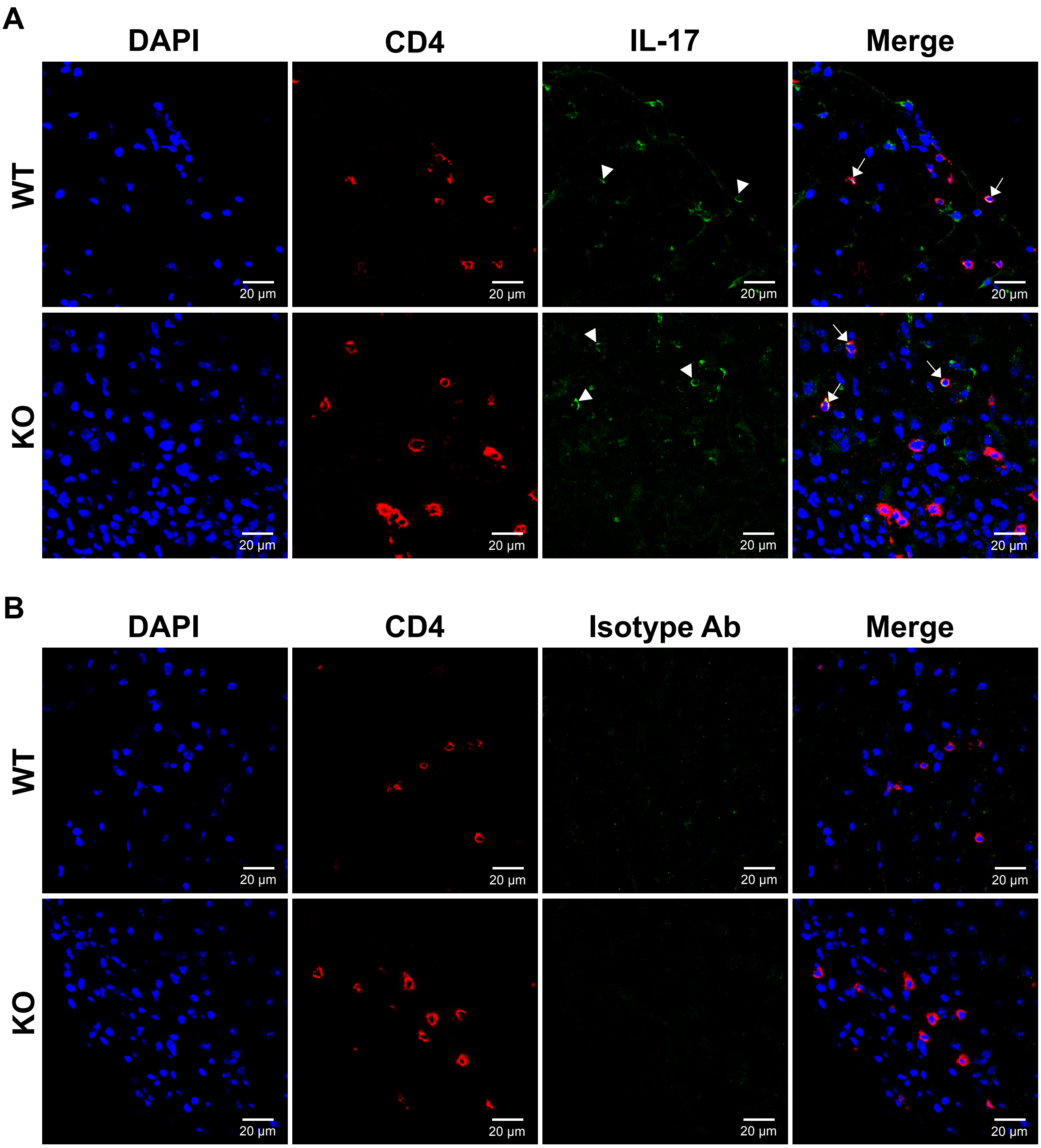

Figure S2. Th17 cells are detected in the spinal cord by immunofluorescence assay. A. Frozen sections of the spinal cord from MOG-immunized WT and CXCR3$^{-/-}$ (KO) mice at peak of disease (day 15) were subjected to immunofluorescence staining with rat anti-mouse CD4 / biotin conjugated-donkey anti-rat / Alexa 555-conjugated streptavidin (red) and goat anti-mouse IL-17 / Alexa 488-conjugated donkey anti-goat (green) followed by counterstaining with DAPI (blue). Arrowheads indicate IL-17^{+} cells and arrows indicate CD4^{+}IL-17^{+} cells (Th17). **B**. Same procedure in A was performed except that goat anti-mouse IL-17 was replaced by isotype control antibody.
