# Supplementary material for: CXCR3 signaling in glial cells ameliorates experimental autoimmune encephalomyelitis by restraining the generation of a pro-Th17 cytokine milieu and reducing CNS-infiltrating Th17 cells
Source: J Neuroinflammation. 2016 Apr 11;13:76. doi: 10.1186/s12974-016-0536-4 (PMC4828793; doi:10.1186/s12974-016-0536-4)
Supplement: Additional file 3: Figure S3. — The gating strategy of FACS analysis to identify subpopulations of CD4+IL-17+ and CD4+IFN-γ+ cells shown in Fig. 4. Mononuclear cells were isolated from the spinal cord of WT and CXCR3-/- recipient mice at day 12 post-immunization and subjected to FACS analysis. Lymphocytes were gated according to forward and side scatter and then subsequently gated on CD4+ cells. CD4+ cells were further gated to analyze IFN-γ+ cells (CD4+IFN-γ+, Th1) and IL-17+ cells (CD4+IL-17+, Th17). Data are representative of three independent experiments. (PDF 226 kb) [file 12974_2016_536_MOESM3_ESM.pdf]

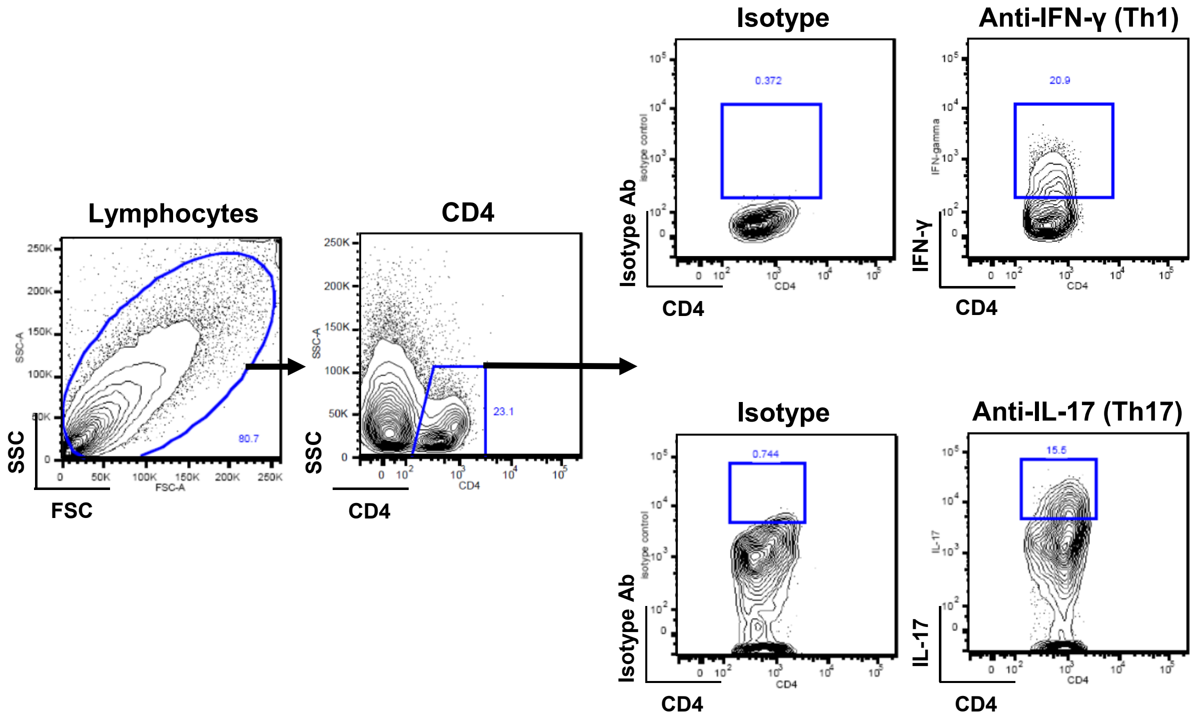

Figure S3. The gating strategy of FACS analysis to identify subpopulations of CD4⁺IL-17⁺ and CD4⁺IFN-γ⁺ cells shown in Fig. 4. Mononuclear cells were isolated from the spinal cord of WT and CXCR3 $^{-/-}$ recipient mice at day 12 post-immunization and subjected to FACS analysis. Lymphocytes were gated according to forward and side scatter and then subsequently gated on CD4⁺ cells. CD4⁺ cells were further gated to analyze IFN-γ⁺ cells (CD4⁺IFN-γ⁺, Th1) and IL-17⁺ cells (CD4⁺IL-17⁺, Th17). Data are representative of three independent experiments.
