# Supplementary material for: CXCR3 signaling in glial cells ameliorates experimental autoimmune encephalomyelitis by restraining the generation of a pro-Th17 cytokine milieu and reducing CNS-infiltrating Th17 cells
Source: J Neuroinflammation. 2016 Apr 11;13:76. doi: 10.1186/s12974-016-0536-4 (PMC4828793; doi:10.1186/s12974-016-0536-4)
Supplement: Additional file 4: Figure S4. — The gating strategy of FACS analysis to identify proliferating Th17 cells shown in Fig. 5. Mononuclear cells were isolated from the spinal cord of MOG-immunized WT and CXCR3-/- recipient mice at day12 post-immunization and subjected to FACS analysis. Lymphocytes were gated according to forward and side scatter and then subsequently gated on CD4+ cells. CD4+ cells were further gated to analyze cell cycle phase distribution. The phases of the cell cycle of CD4 cells were indicated (G0/G1: BrdU- 7-AAD+; G2/M: BrdU- 7-AADhigh; S: BrdU+ 7-AADhigh; Apoptosis: BrdU- 7-AADlow). CD4+ cells in S phase were further gated to analyze IL-17+ cells (CD4+IL-17+, Th17). Data are representative of two independent experiments. (PDF 183 kb) [file 12974_2016_536_MOESM4_ESM.pdf]

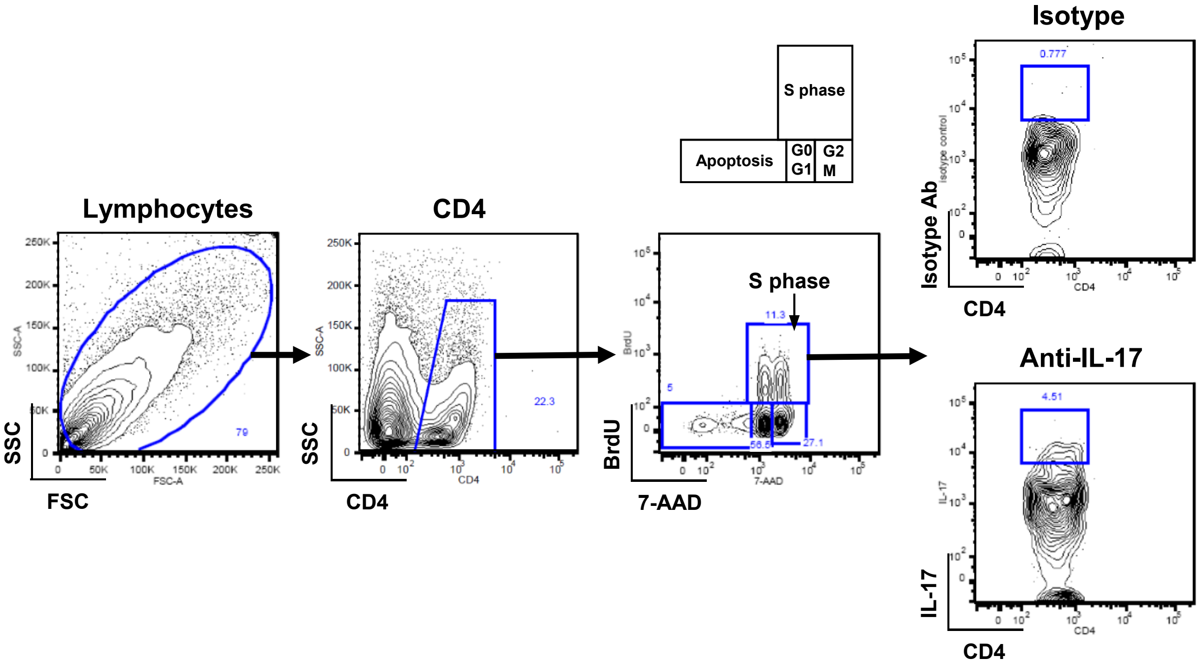

Figure S4. The gating strategy of FACS analysis to identify proliferating Th17 cells shown in Fig. 5. Mononuclear cells were isolated from the spinal cord of MOG-immunized WT and CXCR3 $^{-/-}$ recipient mice at day12 post-immunization and subjected to FACS analysis. Lymphocytes were gated according to forward and side scatter and then subsequently gated on $CD4^+$ cells. $CD4^+$ cells were further gated to analyze cell cycle phase distribution. The phases of the cell cycle of CD4 cells were indicated (G0/G1: $BrdU^-$ $7\text{-}AAD^+$; G2/M: $BrdU^-$ $7\text{-}AAD^{high}$; S: $BrdU^+$ $7\text{-}AAD^{high}$; Apoptosis: $BrdU^-$ $7\text{-}AAD^{low}$). $CD4^+$ cells in S phase were further gated to analyze IL-17^+ cells ($CD4^+$IL-17^+, Th17). Data are representative of two independent experiments.
