# Supplementary material for: CXCR3 signaling in glial cells ameliorates experimental autoimmune encephalomyelitis by restraining the generation of a pro-Th17 cytokine milieu and reducing CNS-infiltrating Th17 cells
Source: J Neuroinflammation. 2016 Apr 11;13:76. doi: 10.1186/s12974-016-0536-4 (PMC4828793; doi:10.1186/s12974-016-0536-4)
Supplement: Additional file 5: Figure S5. — Isotype control antibody for anti-CXCR3 in Fig. 9. Frozen sections were subjected to immunofluorescence staining as describe in Fig. 9 except that anti-CXCR3 was replaced with isotype control antibody. a Astrocytes were marked as GFAP+ cells. b Microglia were marked as Iba1+ cells. GFAP: red, Iba1: red, isotype control antibody for anti-CXCR3: green, DAPI: blue. (PDF 981 kb) [file 12974_2016_536_MOESM5_ESM.pdf]

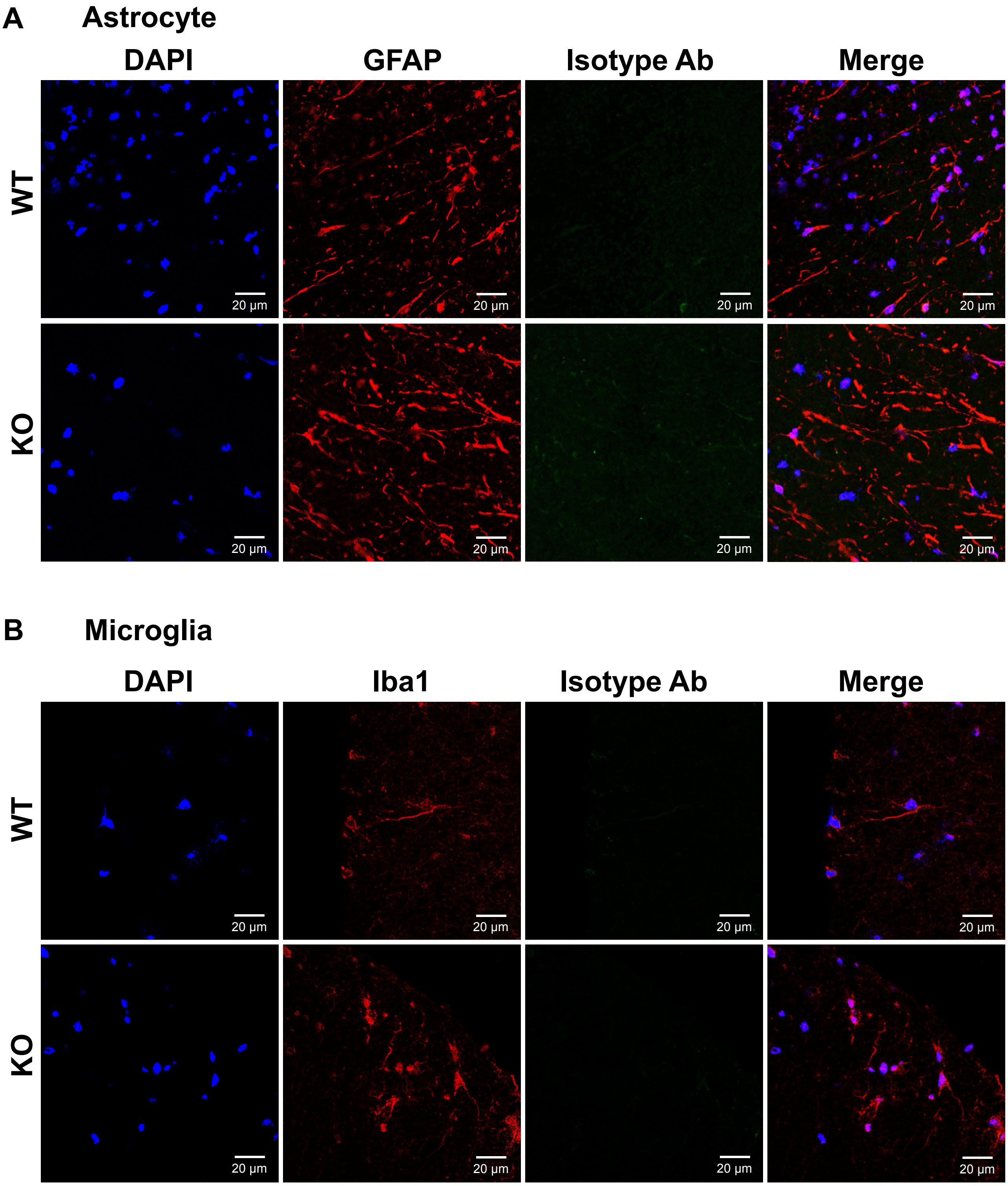

Figure S5. Isotype control antibody for anti-CXCR3 in Fig. 9. Frozen sections were subjected to immunofluorescence staining as describe in Figure 9 except that anti-CXCR3 was replaced with isotype control antibody. **A**. Astrocytes were marked as GFAP$^+$ cells. **B**. Microglia were marked as Iba1$^+$ cells. GFAP: red, Iba1: red, isotype control antibody for anti-CXCR3: green, DAPI: blue.
