# Supplementary material for: CXCR3 signaling in glial cells ameliorates experimental autoimmune encephalomyelitis by restraining the generation of a pro-Th17 cytokine milieu and reducing CNS-infiltrating Th17 cells
Source: J Neuroinflammation. 2016 Apr 11;13:76. doi: 10.1186/s12974-016-0536-4 (PMC4828793; doi:10.1186/s12974-016-0536-4)
Supplement: Additional file 6: Table S1. — The effect of CXCR3 in various studies. (DOCX 17 kb) [file 12974_2016_536_MOESM6_ESM.docx]

| Table S1. The effect of CXCR3 in various studies | | | | |
| --- | --- | --- | --- | --- |
| Studies | Mouse strain and source | Antigen used for EAE induction | Results | Comments |
| Liu et al., J. Immunol. 176:4399, 2006 | CXCR3^-/-^ mice (C57BL/6) were backcrossed to WT (C57BL/6) mice for 11 generations and littermate were used. | MOG_35-55_ emulsified in CFA (400 μg Mycobacterium tuberculosis)/ 200 ng Pertussis toxin (PTX) at day 0 and day 2 post-immunization. | CXCR3^-/-^ mice were susceptible to EAE due to the impaired iNOS expression and increased BBB disruption  (CXCR3: protective role) | The background, breeding and housing of WT and CXCR3^-/-^ mice (C57BL/6) were well controlled.  The antigen used for induction of EAE was MOG_35-55_. |
| Muller et al., J. Immunol. 179:2774, 2007 | CXCR3^-/-^ mice (C57BL/6) were backcrossed for 12 generations to the WT (C57BL/6) mice. | 100 μl MOG35-56 (3 mg/ml) and 100 μl CFA with 4 mg/ml Mycobacterium tuberculosis H37Ra and 500 ng PTX was repeated 2 days post-immunization. | CXCR3^-/-^ mice were susceptible to EAE due to the reduced Treg cells recruitment and their interaction with effector cells.  (CXCR3: protective role) | The background, breeding and housing of WT and CXCR3^-/-^ mice (C57BL/6) were well controlled.  The antigen used for induction of EAE was MOG_35-55_. |
| Kohler et al., Brain pathology 18:504, 2008 | Female SJL/J mice treated with CXCR3 antagonism to block CXCR3. | 50 μg PLP_139-151_ in CFA with 0.5mg/ml Mycobacterium butyricum plus 8,33ng/ml Mycobacterium tuberculosis H37Ra and PTX on days 0 and 2 post-immunization. | Mice treated with CXCR3 antagonism inhibited effector phase of EAE.  (CXCR3: detrimental role) | The mice strain used was SJL/J background. CXCR3^-/-^mice were not used for the study; CXCR3 antagonism was used for blocking CXCR3 signaling.  The antigen used for induction of EAE was PLP_139-151_. |
| Sporici and Isselutz, Eu. J. Immunol. 40:2751 (2010) | Inbred male Lewis rats were treated with anti-CXCR3 to block CXCR3. | An emulsion consisting of guinea pig spinal cord, Mycobacterium butyricum, and mineral oil. | CXCR3 blockade delayed disease onset and inhibited leukocyte infiltration of CNS.  (CXCR3: detrimental role) | The mice strain used was Lewis rats instead of mice. No CXCR3^-/-^ rat was used for the study. anti-CXCR3 antibody was used for blocking CXCR3 function. The antigen used was guinea pig spinal cord, not commonly used antigens-MOG_35-55_ or PLP139_-151_. |
| Lalor and Segal, Eur. J. Immunol. 43:2866 (2013) | CXCR3^-/-^ (C57BL/6) mice were from Dr. C. Gerard and WT mice (C57BL/6) were from NCI (Frederick). | 100 μg MOG_35-55_ containing 4 mg/ml Mycobacterium tuberculosis H37Ra, and 300 ng of PTX at day 0 and day2 post-immunization. | CXCR3^-/-^ (C57BL/6) mice and WT (C57BL/6) mice showed comparable disease incidence, the course, and degree of CNS infiltration.  (CXCR3: no significant effect) | CXCR3^-/-^ (C57BL/6) mice and WT mice (C57BL/6) were from different sources. Whether the CXCR3^-/-^ mice were backcrossed to WT mice for at least 10 generations was not stated in Materials and Methods. Notably, mice from different housing and feeding regimes may yield differences in microbiota, which are known to have a significant impact on immune responses.  MOG_35-55_ was used for induction of EAE. |
| Schmitz et al., Brain, Behavior and Immunity 32:186 (2013) | CXCR3^-/-^ (C57BL/6) mice were bred at the local animal facility; C57BL/6 control mice were purchased from Harlan Winkelmann. | Using the Hooke Kit^TM^ MOG_35-55_/CFA emulsion PTX (EK-0114) containing 100 μg MOG_35-55_ emulsified in 200 μl CFA, and 200 ng PTX in PBS 2 and 24 h post-immunization. | CXCR3^-/-^ mice attenuated autoimmune-mediated pain phenomena.  (CXCR3: detrimental effect) | CXCR3^-/-^ (C57BL/6) mice and WT mice (C57BL/6) were bred in different places.  Whether the CXCR3^-/-^ mice were backcrossed to WT mice for at least 10 generations was not stated in Materials and Methods. Notably, mice from different housing and feeding regimes may yield differences in microbiota, which are known to have a significant impact on immune responses.  MOG_35-55_ was used for induction of EAE.  The antigen used for induction of EAE was MOG_35-55_. |
| Chung and Liao, present study | CXCR3^-/-^ (C57BL/6) mice were backcrossed to C57BL/6 WT mice for 10 generations. | 100 μg of MOG_35-55_ in CFA containing 400 μg of Mycobacterium tuberculosis H37Ra and 200 ng PTX on days 0 and 2 post-immunization. | CXCR3^-/-^ mice were susceptible to EAE with increased Th17 infiltrating in CNS as compared with WT mice.  (CXCR3: protective role) | The background, breeding and housing of WT and CXCR3^-/-^ mice (C57BL/6) were well controlled.  The antigen used for induction of EAE was MOG_35-55_. |
